# *circPARD3* drives malignant progression and chemoresistance of laryngeal squamous cell carcinoma by inhibiting autophagy through the PRKCI-Akt-mTOR pathway

**DOI:** 10.1186/s12943-020-01279-2

**Published:** 2020-11-24

**Authors:** Wei Gao, Huina Guo, Min Niu, Xiwang Zheng, Yuliang Zhang, Xuting Xue, Yunfeng Bo, Xiaoya Guan, Zhongxun Li, Yujia Guo, Long He, Yu Zhang, Li Li, Jimin Cao, Yongyan Wu

**Affiliations:** 1 Shanxi Key Laboratory of Otorhinolaryngology Head and Neck Cancer, First Hospital of Shanxi Medical University, Taiyuan, 030001 China; 2 Shanxi Province Clinical Medical Research Center for Precision Medicine of Head and Neck Cancer, First Hospital of Shanxi Medical University, Taiyuan, 030001 China; 3 Department of Otolaryngology Head & Neck Surgery, First Hospital of Shanxi Medical University, Taiyuan, 030001 China; 4 Key Laboratory of Cellular Physiology, Ministry of Education, Shanxi Medical University, Taiyuan, 030001 China; 5 Department of Cell Biology and Genetics, Basic Medical School of Shanxi Medical University, Taiyuan, 030001 China; 6 Department of Pathology, Shanxi Cancer Hospital, Shanxi Medical University, Taiyuan, 030013 China; 7 Department of Physiology, Shanxi Medical University, Taiyuan, 030001 China; 8 Department of Biochemistry & Molecular Biology, Shanxi Medical University, Taiyuan, 030001 China

**Keywords:** Autophagy, Circular RNA, Chemoresistance, PRKCI, Migration and invasion, PI3K-Akt-mTOR signaling pathway

## Abstract

**Background:**

Laryngeal squamous cell carcinoma (LSCC) is the second most common malignant tumor in head and neck. Autophagy and circular RNAs (circRNAs) play critical roles in cancer progression and chemoresistance. However, the function and mechanism of circRNA in autophagy regulation of LSCC remain unclear.

**Methods:**

The autophagy-suppressive circRNA *circPARD3* was identified via RNA sequencing of 107 LSCC tissues and paired adjacent normal mucosal (ANM) tissues and high-content screening. RT-PCR, Sanger sequencing, qPCR and fluorescence in situ hybridization were performed to detect *circPARD3* expression and subcellular localization. Biological functions of *circPARD3* were assessed by proliferation, migration, invasion, autophagic flux, and chemoresistance assays using in vitro and in vivo models. The mechanism of *circPARD3* was investigated by RNA immunoprecipitation, RNA pulldown, luciferase reporter assays, western blotting and immunohistochemical staining.

**Results:**

Autophagy was inhibited in LSCC, and *circPARD3* was upregulated in the LSCC tissues (*n* = 100, *p* < 0.001). High *circPARD3* level was associated with advanced T stages (*p* < 0.05), N stages (*p* = 0.001), clinical stages (*p* < 0.001), poor differentiation degree (*p* = 0.025), and poor prognosis (*p* = 0.002) of LSCC patients (*n* = 100). Functionally, *circPARD3* inhibited autophagy and promoted LSCC cell proliferation, migration, invasion and chemoresistance. We further revealed that activation of the PRKCI-Akt-mTOR pathway through sponging *miR-145-5p* was the main mechanism of *circPARD3* inhibited autophagy, promoting LSCC progression and chemoresistance.

**Conclusion:**

Our study reveals that the novel autophagy-suppressive *circPARD3* promotes LSCC progression and chemoresistance through the PRKCI-Akt-mTOR pathway, providing new insights into circRNA-mediated autophagy regulation and potential biomarker and target for LSCC treatment.

**Graphical abstract:**

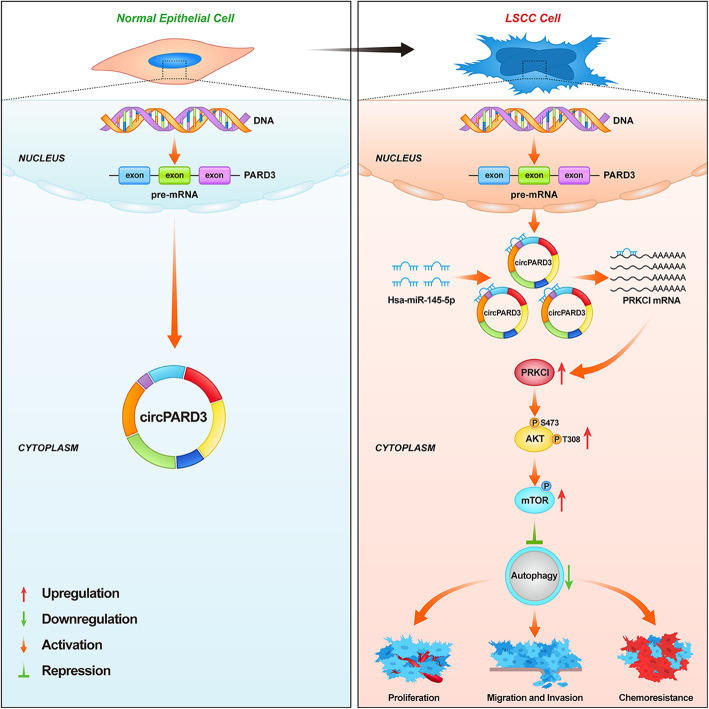

**Supplementary Information:**

The online version contains supplementary material available at 10.1186/s12943-020-01279-2.

## Background

Laryngeal squamous cell carcinoma (LSCC) is the second most common malignant tumor in head and neck, with increasing incidence and mortality [1]. LSCC is prone to local invasion, cervical lymph node metastasis and chemoresistance, which are the main factors leading to poor prognosis in patients [2, 3]. Autophagy is the biological process of eukaryotic cells using lysosomes to degrade their damaged organelles and macromolecular substances. Autophagy is important for cellular metabolism, organelles renewal and intracellular homeostasis maintenance [4]. Recent studies suggested that autophagy plays pivotal role in tumorigenesis, recurrence, metastasis, and chemoresistance [5]. However, the autophagy status and regulatory mechanisms in LSCC remain unclear.

Circular RNAs (circRNAs) are a class of covalently closed circular RNA molecules, which are more stable than linear RNA because they are unaffected by RNA exonuclease. Therefore, circRNAs have unique advantages as markers for disease diagnosis and prognosis [6]. circRNA expression has tissue and cell specificity, playing an important regulatory role in various physiological and pathological processes [7]. circRNAs can function as a microRNA (miRNA) sponge to bind miRNAs specifically. In consequence, it relieves the inhibitory effects of miRNA on downstream target genes, thus upregulating the expression levels of the target genes [8]. Furthermore, some circRNAs exert biological functions via binding to RNA-binding proteins or translating into proteins [9, 10]. Previous studies indicated that circRNAs are involved in regulation of autophagy in several diseases such as breast cancer, sciatic nerve injury, and cerebral ischemic stroke [11–13]. However, the regulatory relationship between circRNAs and autophagy and the clinical significance of autophagy-related circRNAs in diagnosis and treatment of LSCC remain largely unexplored.

In this study, we demonstrated that autophagy is inhibited in LSCC. Strikingly, we identified a novel autophagy suppressive circRNA *circPARD3*, which is significantly upregulated in LSCC tissues. High *circPARD3* expression is significantly associated with malignant progression and poor prognosis of LSCC patients. We found that *circPARD3* promotes LSCC cell proliferation, migration, invasion and chemoresistance by inhibiting autophagy. Our data further revealed that *circPARD3* upregulates PRKCI expression by sponging *miR-145-5p*, thereby activating the Akt-mTOR signaling axis and inhibiting autophagy. These findings extend the understanding of autophagy and circRNA in LSCC progression and chemoresistance, and highlight the significance of *circPARD3* in the regulation of autophagy and chemoresistance in LSCC.

## Methods

### Tumor specimens

Tumor specimens were collected from patients undergoing surgery at the Department of Otolaryngology Head and Neck Surgery, First Hospital of Shanxi Medical University. A total of three cohorts of LSCC specimens were used in this study (Additional file 3: Figure S1). Cohort 1 of 138 LSCC patients with available archived formalin-fixed paraffin-embedded (FFPE) LSCC tissues was used for immunohistochemical staining (IHC) or immunofluorescence (IF). Cohort 2 of 107 LSCC cases with LSCC tissues (*n* = 107) and paired adjacent normal mucosa (ANM) tissues (*n* = 107) was used for RNA sequencing. Cohort 3 of 100 LSCC cases with LSCC tissues and paired ANM tissues was used for qPCR experiments. None of the patients received chemotherapy or radiotherapy before surgery. Clinical features of LSCC samples are shown in Additional file 1: Table S1–3.

### Antibodies and reagents

Antibodies against to Akt (Cat # 4685S), Phospho-Akt (Thr308) (Cat # 2965S), Phospho-Akt (Ser473) (Cat # 4060S), mTOR (Cat # 2983S), Phospho-mTOR (Ser2448) (Cat # 5536S), LC3B (Cat # 3868S), SQSTM1/p62 (Cat # 88588S), E-Cadherin (Cat # 3195S), N-Cadherin (Cat # 13116S), and Vimentin (Cat # 5741S) were purchased from Cell Signaling Technology (Danvers, MA). PRKCI antibody (Cat # 13883–1-AP) was purchased from Proteintech Group, Inc. (Rosemont, IL). GAPDH antibody (Cat # HC301–02) was purchased from TransGen Biotech (Beijing, China). Rapamycin (Cat # S1039), SC79 (Cat # S7863), 3-Methyladenine (3-MA) (Cat # S2767), MK-2206 2HCl (MK-2206) (Cat # S1078), MHY1485 (Cat # S7811), and Actinomycin D (Cat # S8946) were purchased from Selleck Chemicals (Houston, TX). Detailed information of the chemicals is shown in Additional file 1: Table S4.

### Cell culture

Human LSCC cell line Tu 177 (purchased from Bioleaf Biotech Corporation, Shanghai, China), human oral keratinocytes (HOK) (obtained from ScienCell Research Laboratories, Carlsbad, CA), HEK293T cells (obtained from the China Center for Type Culture Collection), human LSCC cell line FD-LSC-1 (kindly provided by Professor Liang Zhou [14]), and human normal lung fibroblast cell line MRC-5 (purchased from the China Center for Type Culture Collection) were cultured in a humidified atmosphere at 37 °C with 5% CO2. Cell lines were confirmed to be mycoplasma free using the TransDetect PCR Mycoplasma Detection Kit (TransGen Biotech, Beijing, China). For half-life analysis of RNA, transcription was inhibited using Actinomycin D (2 μg/mL). Detailed information for cell lines and media is shown in Additional file 1: Table S5.

### RNA and genomic DNA (gDNA) extraction, RNase R treatment, and quantitative real time PCR (qPCR)

Total RNA was extracted from cells and tumor samples using Trizol reagent (Invitrogen, Waltham, MA) according to the manufacturer’s instructions. Cytoplasmic and nuclear RNA was isolated using a PARIS kit (ThermoFisher Scientific, Waltham, MA). gDNA was extracted using a genomic DNA isolation kit (TIANGEN Biotech (Beijing) Co., Ltd., Beijing, China). For RNase R treatment, 2 μg of total RNA was incubated for 10 min at 37 °C with or without 3 U/μg RNase R (Geneseed Biotech Co., Ltd., Guangzhou, China). For PCR or qPCR analysis of mRNA and circRNA, cDNA was synthesized using a HiScript II 1st Strand cDNA Synthesis Kit (Vazyme, Nanjing, China). For qPCR of miRNA, cDNA was synthesized using an All-in-One miRNA First-Strand cDNA Synthesis Kit (GeneCopoeia, Rockville, MD). qPCR was performed using PerfectStart Green qPCR SuperMix (TransGen Biotech) on LightCycler 96 Instrument (Roche, Basel, Switzerland). The relative RNA expression levels were calculated using the 2 ^(−△△CT)^ method. The *18 s* rRNA (for circRNA and mRNA) and *U6* small nuclear RNA were used as internal controls. Primer sequences are shown in Additional file 1: Table S6.

### RNA sequencing

The RNA integrity was examined with Bioanalyzer 2100 (Agilent, Santa Clara, CA). The RNA samples of 107 LSCC and paired ANM tissue with RIN > 7 were subjected to library construction, and then sequenced on Illumina HiSeq 4000 (circRNA and mRNA) or HiSeq 2000 (miRNA). RNA sequencing technology was provided by Novogene (Beijing, China). Differentially expressed circRNAs, miRNAs, and mRNAs were analyzed as previous reported [15] (Additional file 1: Table S7–9).

### Plasmid construction

The *circPARD3* overexpression plasmid (circPARD3-OE) was generated by inserting the full-length of *circPARD3* sequence into the pLO5-ciR lentiviral vector (Geneseed Biotech Co., Ltd). shRNA lentiviral plasmid targeting *circPARD3* (sh-circPARD3) was constructed by inserting annealed shRNA template DNA sequence into the pLKO.1 vector. The PRKCI overexpression plasmid (PRKCI-OE) was generated by inserting the *PRKCI* ORF sequence into the pLenti-puro vector (a gift from Ie-Ming Shih, Addgene plasmid # 39481 [16]). To construct luciferase reporter plasmids, the sequences of wild-type *circPARD3*, *miR-145-5p* binding site mutated *circPARD3,* wild-type *PRKCI* 3′ UTR, and *miR-145-5p* binding site mutated *PRKCI* 3′ UTR were cloned into the psiCHECK-2 vector (Promega, Madison, WI), respectively.

### siRNAs, miRNA mimics, and inhibitor

siRNAs targeting *circPARD3* (si-circ*-*1: sense: 5′-CACCUGUAGACAGGUAAAUAC-3′, antisense: 5′- GUAUUUACCUGUCUACAGGUG-3′; si-circ*-*2: sense: 5′-CUGUAGACAGGUAAAUACCAG-3′, antisense: 5′- CUGGUAUUUACCUGUCUACAG-3′), siRNAs targeting *PRKCI* (si-PRKCI: sense: 5′-CCGGGUAAUAGGAAGAGGAAGUUAU-3′, antisense: 5′-AUAACUUCCUCUUCCUAUUACCCGG-3′), negative control siRNAs (si-NC), *miR-145-5p* mimics, *miR-145-5p* inhibitor, and NC mimics/inhibitor were synthesized by GenePharma (Shanghai, China). RNA molecule was modified by 2′-OMe to enhance stability. Transfection efficiency of siRNAs, miRNA mimics, and inhibitor was verified by transfecting cells with Cy3 labeled negative control siRNAs (si-NC-Cy3), miRNA mimics (NC mimics-Cy3), and inhibitor (NC inhibitor-Cy3) (Additional file 3: Figure S2).

### Screening of autophagy related circRNA by high-content platform based autophagic flux analysis

To screen autophagy related circRNAs in LSCC, siRNAs targeting the top 15 upregulated circRNAs in the LSCC samples compared to their paired ANM tissues were designed and synthesized by GenePharma (Shanghai, China). FD-LSC-1 and Tu 177 cells stably expressing LC3B-GFP were transfected with siRNAs or si-NC for 24 h, then cells were seeded into 48-well plates and incubated for additional 24 h. Cells were stained with DAPI and were imaged on a PerkinElmer Operetta CLS high-content analysis system (Waltham, MA). Autophagy level was assessed by comparing the amount of green LC3 dots between NC group and specific circRNA knockdown groups.

### Autophagic flux detection in cells

A tandem mCherry-EGFP-tagged LC3B was used to monitor autophagic flux in cells as previous reported [17]. Briefly, FD-LSC-1 and Tu 177 cells were transduced with mCherry-EGFP-LC3B lentiviruses for 48 h, then mCherry-EGFP-LC3B expressing cells were screened by puromycin. For autophagic flux detection, mCherry-EGFP-LC3B expressed cells were examined by Leica TCS SP8 confocal laser scanning microscope (Leica Microsystems Inc.). The number of GFP and mCherry puncta per cell was quantified manually. At least 10 cells in each of three independent experiments were analyzed randomly.

### Bioinformatics analysis

Gene Set Enrichment Analysis (GSEA) [18] was conducted to explore differentially expressed mRNA involved gene sets. Kyoto Encyclopedia of Genes and Genomes (KEGG) pathway analysis was performed using the clusterProfiler [19] to identify differentially expressed mRNA enriched signaling pathways. Target gene prediction of *hsa-miR-145-5p* was performed using the Targetminer program [20] (http://www.isical.ac.in/~bioinfo_miu/targetminer20.htm). *circPARD3* binding miRNA was predicted using the CircInteractome program [21] (https://circinteractome.nia.nih.gov/mirna_target_sites.html).

### circRNA pulldown

circRNA pulldown was performed using Biotin-labeled *circPARD3* probes (Sangon Biotech, Shanghai, China). Briefly, FD-LSC-1 cells were lysed by IP lysis buffer supplemented with 1 × protease inhibitor and 1 U/μL RNase inhibitor (ThermoFisher Scientific, Waltham, MA) on ice for 30 min with occasional mixing. The lysate was sonicated for 2 min with 10 s on/off cycles. Then the lysate was precleared with 30 μL streptavidin agarose beads (Cat # S1638, Merck KGaA, Darmstadt, Germany) for 1 h at 4 °C with rotation. Biotin-labeled *circPARD3* probes or the negative control (NC) probes were added to the precleared lysate, and the mixtures were incubated at 4 °C overnight. Next, the mixture was incubated with 30 μL streptavidin agarose beads at 37 °C for 1 h. Wash beads-probe-protein complex with 1 mL IP lysis buffer for six times, and then total RNA bound to the beads was extracted, followed by reverse transcription and qPCR detection of *circPARD3* and miRNAs.

### Xenograft tumorigenesis

Animal experiments were approved by the Animal Experimental Research Ethics Committee of Shanxi Medical University and conducted according to its guidelines. BALB/C nude mice (female, 6 weeks) were obtained from Vital River Laboratory Animal Technology Co., Ltd. (Beijing, China) and housed in isolation and ventilation cages (TECNIPLAST S.p.A., Italy) under SPF (Specific pathogen Free) conditions in a climate-controlled room (25 ± 1.5 °C) with 12 h light/dark illumination cycle and 50 ± 10% humidity. To construct xenograft tumor models, 200 μL serum-free DMEM containing 1 × 10^7^ FD-LSC-1 cells were subcutaneously injected into the right flank of each mouse. From day 13, mice were randomized into two groups and intraperitoneally injected with 1 mg/kg Cisplatin (CDDP dissolved in normal Saline, 100 μL) or equal volume of normal Saline (NS) every 2 days. Tumor volume was calculated using the following formula: V (volume) = (length × width^2^)/2. Mice were sacrificed at day 33 and tumors were dissected, weighed, and resected for qPCR, HE staining, IHC staining, and immunofluorescence analysis.

More detailed information of methods could be obtained in Additional file 2: Supplemental Materials and Methods.

### Statistical analysis

Statistical analysis was performed using GraphPad Prism 8 software (San Diego, CA). Comparisons between two groups were conducted by the two-tailed Student’s t-test. Kaplan–Meier method was used to analyze the survival probability of LSCC patients with different expression levels of p62 or *circPARD3*. Data were reported as mean ± SD (standard deviation). *P* values of < 0.05 were considered statistically significant.

## Results

### Autophagy inhibition is associated with malignant progression and poor prognosis of LSCC

**Fig. 1:**
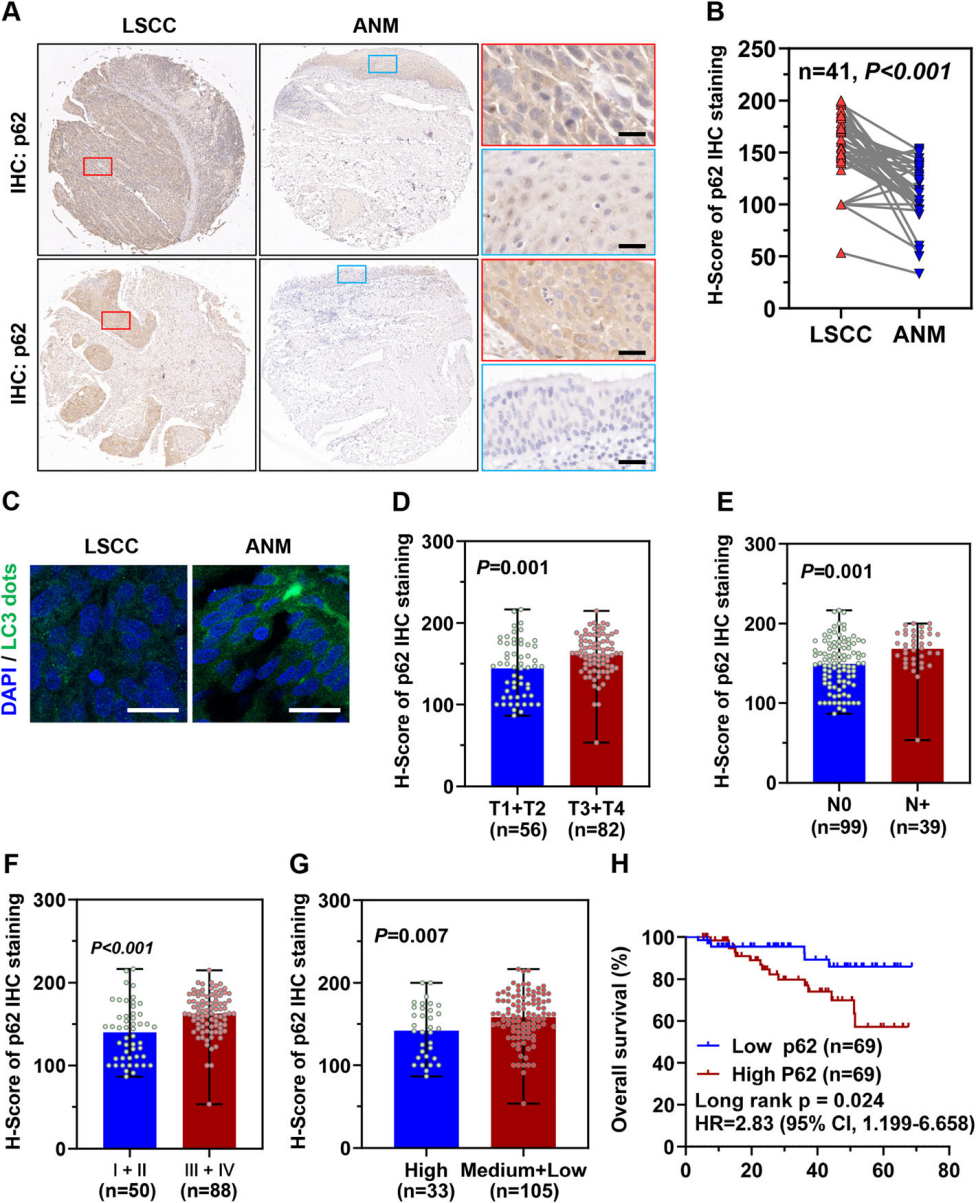
High p62 level is associated with malignant progression and poor prognosis of LSCC patients. Protein levels of p62 in 138 LSCC tissues and 41 ANM tissues (Cohort 1) were determined by IHC staining. **a** Representative image of p62 IHC staining in LSCC tissues and paired ANM tissues. Scale bar, 20 μm. **b** IHC staining results of p62 in 41 paired LSCC tissues and ANM tissues were quantified with CaseViewer version 2.3 and presented as H-Score. **c** Representative confocal microscopy images of LC3B dots (green) in formalin-fixed paraffin-embedded LSCC tissues and paired ANM tissues, nuclei were stained with DAPI (blue). Scale bar, 20 μm. **d-g** Differential expression of p62 protein in 138 LSCC tissues with different T stages (**d**), N stages (**e**), clinical stages (**f**), and pathological differentiation degrees (**g**). The difference of H-score of p62 between two groups was analyzed by Two-tailed Student’s t-test. **h** Kaplan-Meier analysis of the association between p62 protein levels and overall survival in 138 LSCC patients. Expression levels of p62 were divided into high and low subgroups according to the median H-score of IHC staining

p62 (also known as SQSTM1) is an autophagic substrate that is degraded when autophagy is activated and accumulates when autophagy is decreased or inhibited [22]. To determine the autophagy levels of LSCC, we compared p62 protein expression level between LSCC tissues and paired adjacent normal mucosal (ANM) tissues via IHC staining. Results showed that p62 level in the LSCC tissues was significantly higher than that in the ANM tissues (Fig. 1a and b). Furthermore, immunofluorescence staining showed that LSCC tissues contained significantly fewer LC3 dots than that in ANM tissues (Fig. 1c). Analysis of the relationship between p62 level and clinicopathological parameters in 138 LSCC samples indicated that high p62 level was positively correlated with T stages, cervical lymph node metastasis (N stages) and clinical stages (Fig. 1d-f). Moreover, LSCC tissues with low and medium differentiation degree showed higher p62 level than that of their highly differentiated counteracts (Fig. 1g). Kaplan-Meier analysis showed that LSCC patients with high p62 level had shorter overall survival (Fig. 1h). Taken together, autophagy inhibition is an important molecular trait in LSCC and may play important roles in LSCC tumorigenesis and progression.

### Identification of the novel autophagy suppressive circRNA *circPARD3* in LSCC

**Fig. 2:**
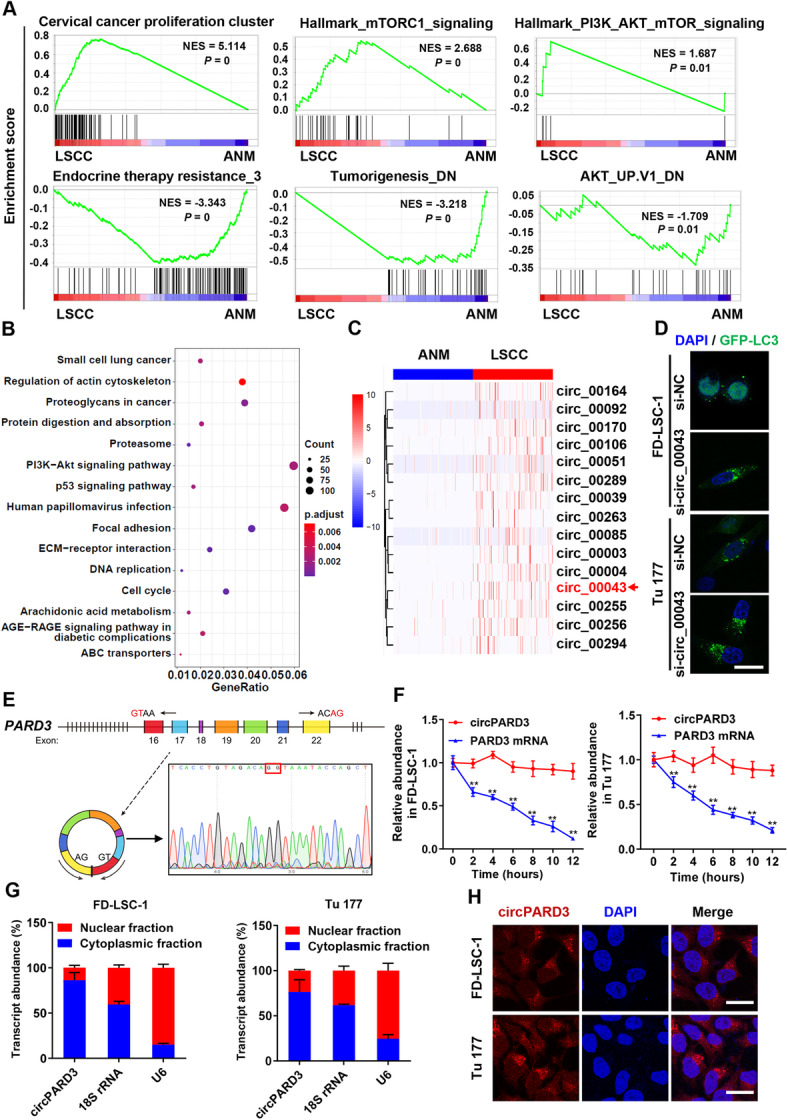
Identification of the autophagy suppressive circRNA *circPARD3* in LSCC. **a-c** RNA sequencing was performed on 107 LSCC tissues and paired ANM tissues (Cohort 2) to screen differentially expressed circRNAs, mRNAs, and miRNAs. GSEA analysis of differentially expressed mRNAs (**a**). NES, normalized enrichment score. KEGG pathway analysis of differentially expressed mRNAs (**b**). Top 15 pathways were plotted according to enriched gene ratio and *p* value. **c** Hierarchical clustering of circRNA differential expression profiles between 107 LSCC tissues and 107 paired ANM tissues. The heatmap was generated from the top 15 differentially expressed circRNAs. **d** FD-LSC-1 and Tu 177 cells expressing LC3B-GFP were transfected with siRNAs or si-NC for 24 h, then cells were seeded into 48-well plates and incubated for additional 24 h. LC3 dots were analyzed on high-content screening system. Representative images of si-circ_00043 transfected cells were shown. Scale bar, 20 μm. **e** Schematic diagram of the genomic location and splicing pattern of *circPARD3* is shown (Left). Validation of the head-to-tail splicing of *circPARD3* by RT-PCR and Sanger sequencing (Right). **f** The relative RNA levels of *circPARD3* and linear *PARD3* mRNA in FD-LSC-1 and Tu 177 cells treated with actinomycin D at the indicated time points were determined by qPCR. **g** *circPARD3* levels in cytoplasm and nucleus of FD-LSC-1 and Tu 177 cells were determined by qPCR analysis. 18S and U6 RNA were used as positive controls of in the cytoplasm and nucleus, respectively. **h** RNA fluorescence in situ hybridization for *circPARD3* was performed in FD-LSC-1 and Tu 177 cells. Nuclei were stained with DAPI (blue) and *circPARD3* probes were labeled with Cy3 (red). Scale bar, 20 μm. Error bars (**f** and **g**) represent SD of three independent experiments. **P < 0.01

RNA sequencing on 107 LSCC tissues and paired ANM tissues found 304 differentially expressed circRNAs in the LSCC tissues, in which 189 upregulated and 115 downregulated (Additional file 1: Table S7). We also found 111 miRNAs with increased expression, and 75 miRNAs with decreased expression, 2467 mRNAs with increased expression, and 2140 mRNAs with decreased expression in LSCC tissues (Additional file 1: Table S8 and 9). GSEA analysis revealed that the differentially expressed genes were mainly involved in cancer cell proliferation, PI3K-Akt-mTOR, therapy resistance, and tumorigenesis (Fig. 2a). KEGG pathway analysis confirmed that the differentially expressed genes were mainly enriched in the PI3K-Akt, cell cycle, focal adhesion and other signaling pathways (Fig. 2b).

LC3B (Microtubule Associated Protein 1 Light Chain 3 Beta) is a recognized autophagic marker [23]. To identify autophagy related circRNAs, the top 15 circRNAs upregulated in LSCC tissues (Fig. 2c) were knockdown individually using siRNAs in LC3B-GFP expressing LSCC cell lines FD-LSC-1 and Tu 177. High-content screening showed that LC3 dots were significantly increased in cells with circ_00043 knockdown (Fig. 2d), indicating that circ_00043 inhibited autophagy of LSCC cells. Sequence analysis indicated that circ_00043 is formed by backsplicing of exons 16–22 of human *PARD3* mRNA (Fig. 2e), hence, we named it as *circPARD3*. The head-to-tail circular structure of *circPARD3* was verified by RT-PCR and Sanger sequencing (Fig. 2e). Furthermore, RT-PCR using back-to-back primers demonstrated that the head-to-tail structure of *circPARD3* was formed by backsplicing of the *PARD3* mRNA rather than by genomic rearrangement (Additional file 3: Figure S3A).

LSCC cells were treated with actinomycin D, results showed that the half-life of *circPARD3* was significantly longer than that of linear *PARD3* mRNA (Fig. 2f). Furthermore, the total RNA purified from LSCC cells was treated with RNase R, and we found that the linear *PARD3* mRNA was decreased after RNase R treatment, while *circPARD3* was not significantly degraded, indicating that *circPARD3* is resistant to RNase R (Additional file 3: Figure S3B). After separating the nucleus and cytoplasm of FD-LSC-1 and Tu 177 cells, qPCR showed that *circPARD3* mainly existed in the cytoplasm (Fig. 2g), which was further confirmed by RNA fluorescence in situ hybridization (Fig. 2h). Collectively, our study identified *circPARD3* as an autophagy suppressive circRNA in LSCC, and we further verified its structural characteristics.

### Upregulation of *circPARD3* is associated with malignant progression and poor prognosis of LSCC

**Fig. 3:**
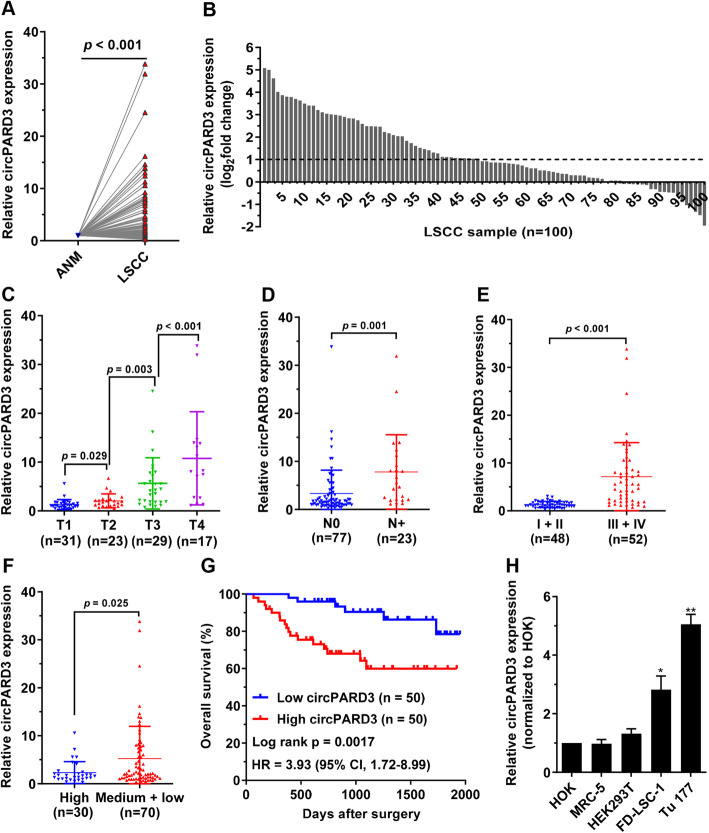
Upregulation of *circPARD3* and its clinical significance in LSCC. The relative RNA levels of *circPARD3* in 100 LSCC tissues and paired ANM tissues (Cohort 3) were determined by qPCR. **a** Comparison of *circPARD3* levels between LSCC tissues and paired ANM tissues (*n* = 100). Two-tailed Student’s t-test. **b** The relative expression levels of *circPARD3* in 100 LSCC tissue samples were normalized to paired ANM tissues. **c-f** Association analysis between *circPARD3* levels and T stages (**c**), N stages (**d**), clinical stages (**e**), and pathological differentiation degrees (**f**). The difference of *circPARD3* levels between two groups was analyzed by Two-tailed Student’s t-test. Error bars represent SD**. g** Kaplan-Meier analysis of the association between *circPARD3* levels and overall survival of 100 LSCC patients. Expression levels of *circPARD3* were divided into high and low subgroups according to the median relative RNA abundance of qPCR experiments. **h** The relative expression of *circPARD3* in LSCC cell lines (FD-LSC-1, Tu 177) and normal control cell lines (HOK, MRC-5, HEK293T) was determined by qPCR analysis. Error bars represent SD of three independent experiments. *P < 0.05, **P < 0.01

Next, we examined the expression level of *circPARD3* in 100 LSCC tissues and paired ANM tissues. qPCR results showed that *circPARD3* level in the LSCC tissues was significantly higher than that in the ANM tissues (Fig. 3a) in up to 80% of paired samples (Fig. 3b). Furthermore, high *circPARD3* level was positively correlated with T stags, cervical lymph node metastasis (N stages) and clinical stages (Fig. 3c-e). Notably, the expression levels of *circPARD3* increased progressively from T1 to T4 stage (Fig. 3c). Moreover, *circPARD3* level was higher in lowly differentiated LSCC tissues than that in highly differentiated tissues (Fig. 3f). However, the *circPARD3* level was not significantly correlated with sex, age, smoking or alcohol consumption in LSCC patients (*p* > 0.05). We divided the LSCC tissues into the high-expression (*n* = 50) and low-expression (*n* = 50) groups according to median *circPARD3* expression level. Kaplan-Meier analysis showed that patients with high *circPARD3* expression had much shorter survival (Fig. 3g). We also compared *circPARD3* expression between LSCC cell lines (FD-LSC-1 and Tu 177) and normal control cell lines (HOK, MRC-5, and HEK293T). The results showed that *circPARD3* expression was significantly higher in LSCC cells than control cells (Fig. 3h). Taken together, these data indicated that upregulation of *circPARD3* is closely associated with malignant progression of LSCC, therefore *circPARD3* may play an oncogenic role in LSCC.

### *circPARD3* inhibits autophagy of LSCC cells

**Fig. 4:**
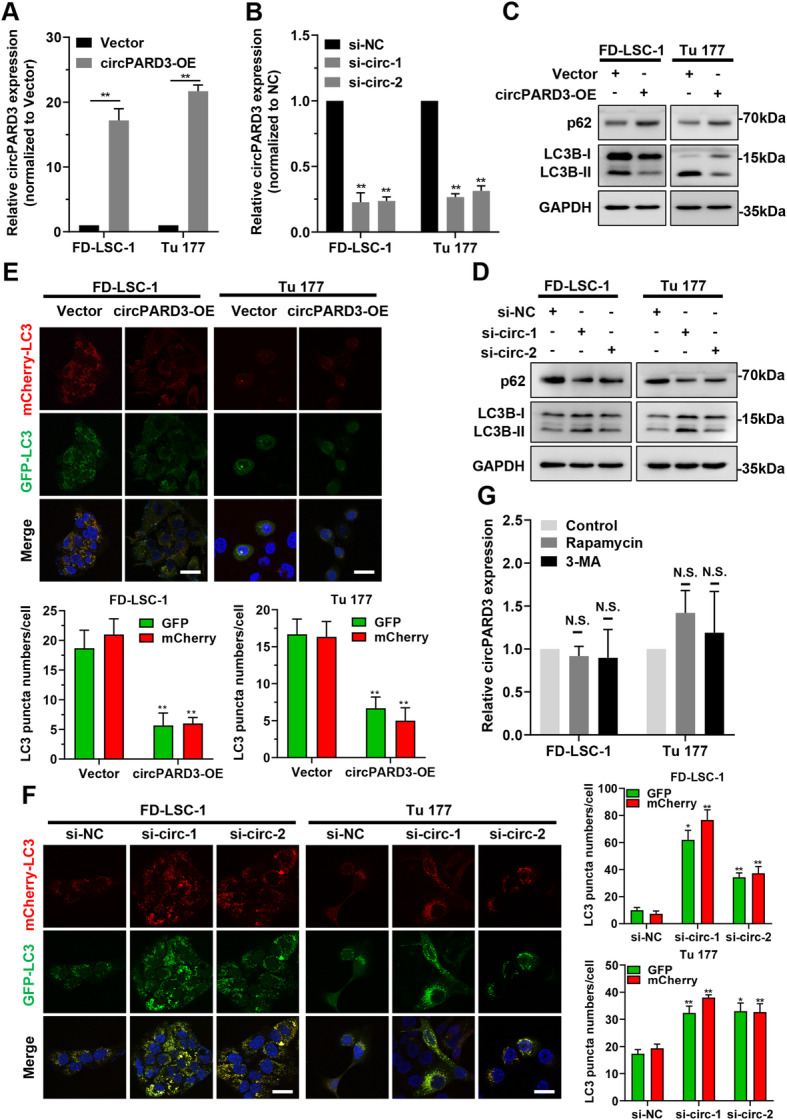
*circPARD3* inhibits autophagy of LSCC cells. **a** FD-LSC-1 and Tu 177 cells were infected with *circPARD3* overexpression lentiviruses (circPARD3-OE) or control empty vector (Vector). Expression of *circPARD3* was examined by qPCR. **b** FD-LSC-1 and Tu 177 cells were transfected with siRNAs targeting *circPARD3* (si-circ-1 and si-circ-2) or NC siRNAs (si-NC) for 48 h. Expression of *circPARD3* was examined by qPCR. **c** and **d** FD-LSC-1 and Tu 177 cells were infected with *circPARD3* overexpression lentiviruses or transfected with siRNAs targeting *circPARD3.* Western blotting was performed to detect the expression of LC3B and p62 after overexpression (**c**) or silencing of *circPARD3* (**d**). **e** and **f** mCherry-EGFP-LC3B labeled FD-LSC-1 and Tu 177 cells were infected with *circPARD3* overexpression lentiviruses or transfected with siRNAs targeting *circPARD3.* Autophagic flux was analyzed by confocal microscopy after overexpression (**e**) or silencing (**f**) of *circPARD3*. Scale bar, 25 μm. **g** qPCR analysis of *circPARD3* in FD-LSC-1 and Tu 177 cells after Rapamycin (100 nM for 24 h) or 3-Methyladenine (3-MA, 2.5 mM for 24 h) treatment. Error bars represent SD of three independent experiments. *P < 0.05, **P < 0.01. N.S., no significant

To validate that *circPARD3* regulates autophagy in LSCC, we transfected LSCC cells with *circPARD3* overexpression plasmid and siRNAs, respectively. qPCR results demonstrated that *circPARD3* expression in FD-LSC-1 and Tu 177 cells transfected with *circPARD3* overexpression plasmid and siRNAs was increased and decreased correspondingly (Fig. 4a and b), but linear *PARD3* mRNA did not change significantly (Additional file 3: Figure S4). Detection of autophagy-related markers revealed that LC3-II level was decreased in FD-LSC-1 and Tu 177 cells overexpressing *circPARD3*, but the level of the autophagy substrate protein p62 was increased (Fig. 4c). Conversely, *circPARD3* knockdown led to increased LC3-II level but decreased p62 level (Fig. 4d). Next, we transfected mCherry-GFP-LC3B labeled LSCC cells with *circPARD3* overexpression plasmid or siRNAs, and analyzed the effect of *circPARD3* on autophagy flux by observing fluorescence changes. The numbers of green and red LC3 puncta were decreased in LSCC cells with circPARD3 overexpression (Fig. 4e). By contrast, the numbers of green and red LC3 puncta were increased after *circPARD3* knockdown (Fig. 4f). These results confirmed that *circPARD3* inhibits autophagy of LSCC cells.

To further clarify the relationship between autophagy and *circPARD3* expression, LSCC cells were treated with the autophagy activator Rapamycin, or autophagy inhibitor 3-Methyladenine (3-MA). Results showed that *circPARD3* expression did not significantly differ in FD-LSC-1 and Tu 177 cells after activation or inhibition of autophagy (Fig. 4g). Collectively, *circPARD3* acted as an upstream regulator to inhibit autophagy in LSCC cells, but autophagy level changes did not affect *circPARD3* expression.

### *circPARD3* promotes LSCC cell proliferation, migration, invasion and chemoresistance by inhibiting autophagy

**Fig. 5:**
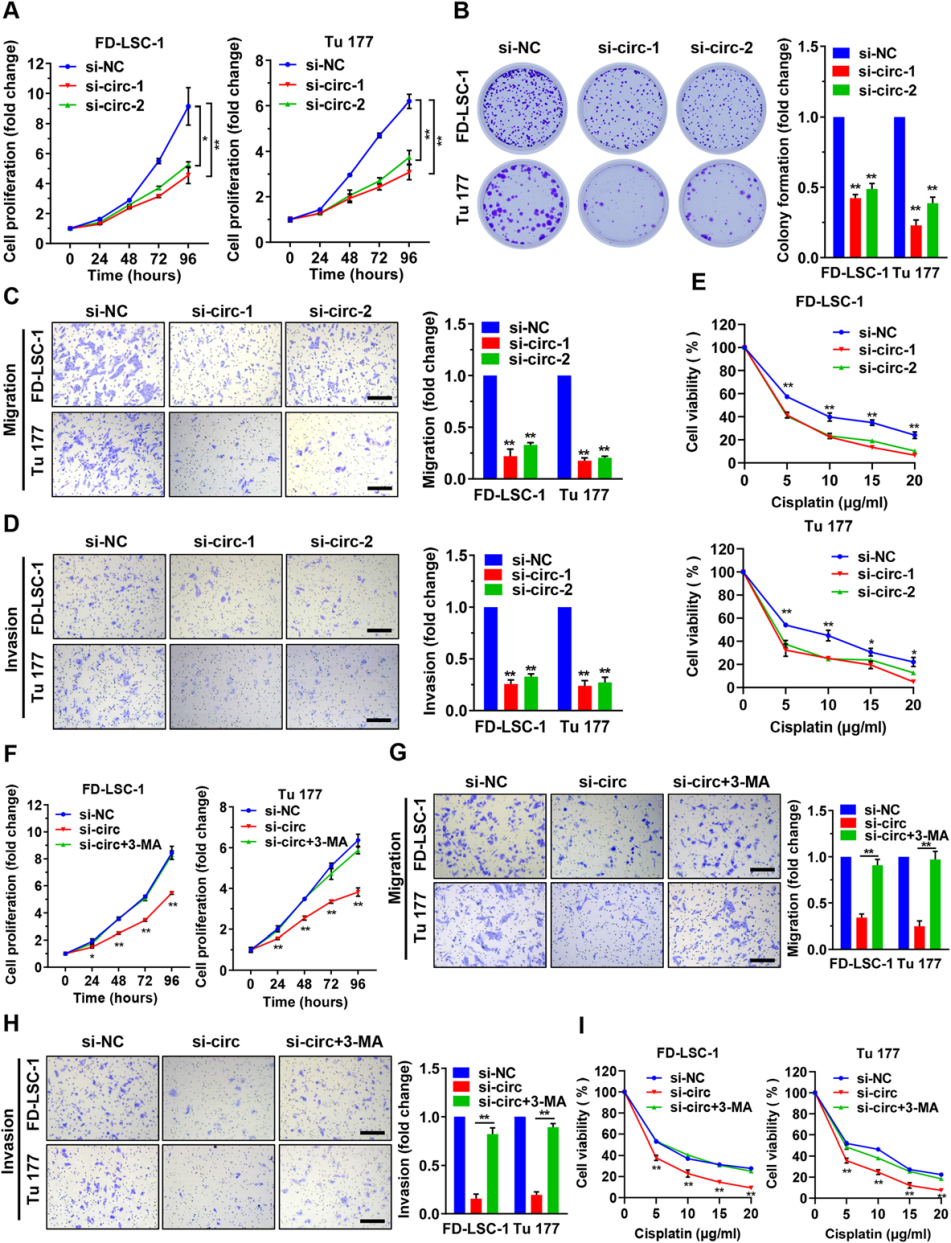
*circPARD3* promotes LSCC cell proliferation, migration, invasion and chemoresistance via inhibiting autophagy. **a** and **b** FD-LSC-1 and Tu 177 cells were transfected with siRNAs targeting *circPARD3* (si-circ-1, si-circ-2) or si-NC, then cell proliferation was determined by CCK8 (**a**) and colony formation assays (**b**). **c** and **d** The migration (**c**) and invasion (**d**) abilities of FD-LSC-1 and Tu 177 cells after silencing of *circPARD3* were detected by Transwell assays. Scale bar, 200 μm. **e** FD-LSC-1 and Tu 177 cells were transfected with siRNAs targeting *circPARD3* or si-NC for 24 h, and then treated with various concentration of Cisplatin for another 24 h. Cell viability was detected by CCK8 assays. **f** FD-LSC-1 and Tu 177 cells were transfected with si-*circPARD3* (si-circ) or si-NC for 24 h, and then treated with 3-MA (2.5 mM). Cell proliferation was determined by CCK8 assays. **g**-**i** FD-LSC-1 and Tu 177 cells were transfected with si-*circPARD3* or si-NC for 24 h, then treated with 3-MA (2.5 mM) for additional 24 h. The migration (**g**) and invasion (**h**) abilities of FD-LSC-1 and Tu 177 cells after various treatments were detected by Transwell assays. Scale bar, 200 μm. Cell viability of LSCC cells treated with various concentrations of Cisplatin for 24 h was determined by CCK8 assays (**i**). Error bars represent SD of three independent experiments. *P < 0.05, **P < 0.01

CCK8 analysis showed that FD-LSC-1 and Tu 177 cell viability decreased after *circPARD3* knockdown (Fig. 5a). Colony formation experiments exhibited that knockdown of *circPARD3* decreased the clonogenic capacity of LSCC cells (Fig. 5b). Furthermore, knockdown of *circPARD3* significantly reduced the migration and invasion abilities of FD-LSC-1 and Tu 177 cells (Fig. 5c and d), and increased the sensitivity of LSCC cells to the chemotherapeutic drug Cisplatin (Fig. 5e). These results demonstrated that *circPARD3* acts as an oncogene to promote LSCC cell proliferation, migration, invasion and chemoresistance.

To determine whether *circPARD3* affects the malignant phenotypes of LSCC cell by regulating autophagy, LSCC cells were transfected with si-circPARD3 and treated with the autophagy inhibitor 3-MA simultaneously. We found that 3-MA treatment reversed the effects of *circPARD3* knockdown on FD-LSC-1 and Tu 177 cell proliferation, migration, invasion and chemosensitivity (Fig. 5f-i). Collectively, these data indicated that *circPARD3* promotes LSCC cell proliferation, migration, invasion and chemoresistance by inhibiting autophagy.

### *circPARD3* acts as a sponge of the tumor suppressor *miR-145-5p* in LSCC

**Fig. 6:**
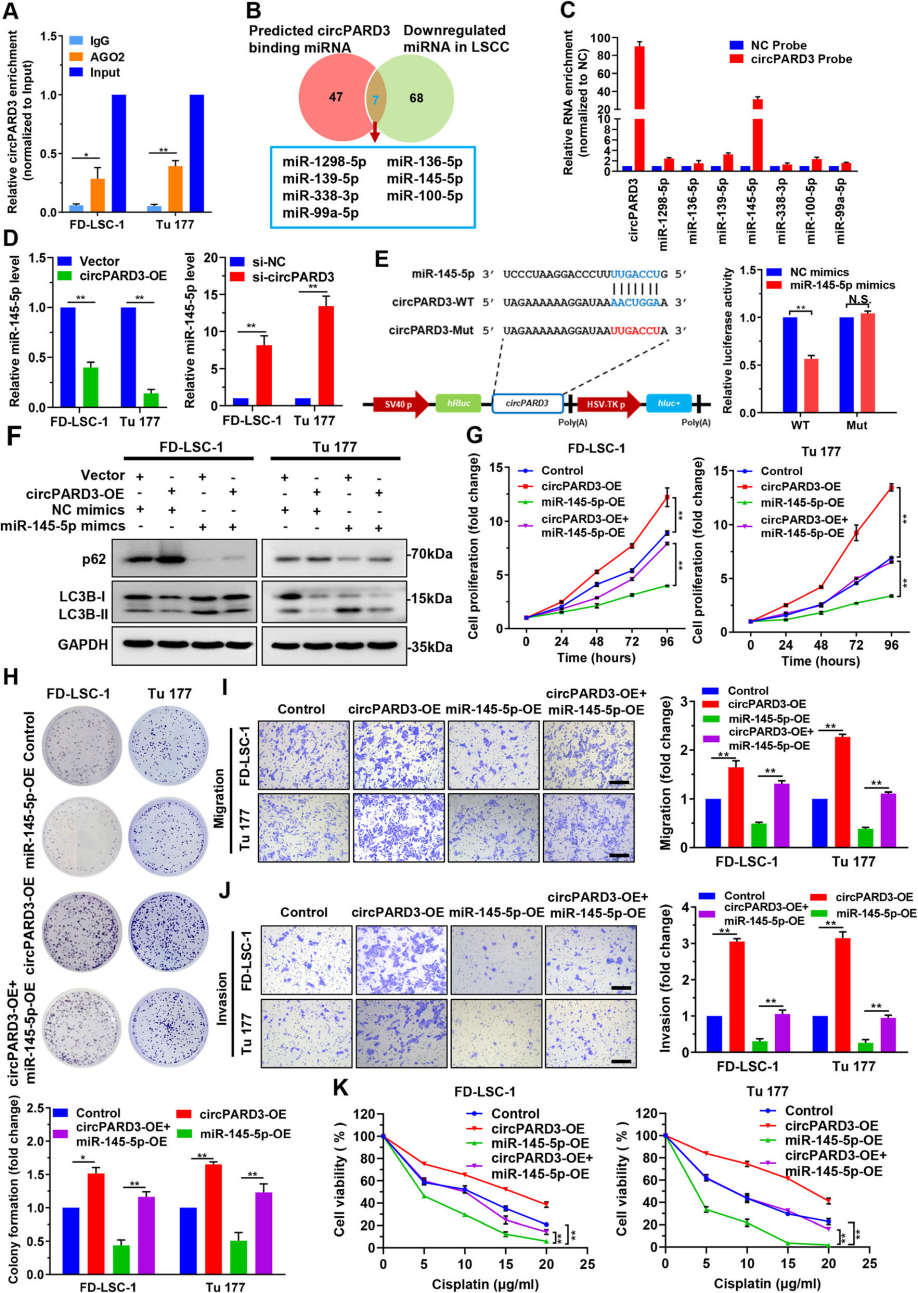
*circPARD3* functions as a sponge of miR-145-5p in LSCC cells. **a** RIP assays were performed using an antibody against AGO2 with extracts from FD-LSC-1 and Tu 177 cells. Enrichment of *circPARD3* in RNA samples after RIP assays was determined by qPCR analysis. **b** Venn diagram shows the intersection of predicted *circPARD3* binding miRNAs and miRNAs downregulated in 107 LSCC tissues from the RNA sequencing data. **c** RNA pulldown assay was performed using Biotin-labeled *circPARD3* probes in FD-LSC-1 cells, then the enrichment of the indicated miRNAs was detected by qPCR analysis. **d** Expression of *miR-145-5p* in LSCC cells overexpressing or silencing of *circPARD3* was determined by qPCR analysis. **e** Luciferase reporter assay of FD-LSC-1 cells cotransfected with *miR-145-5p* mimics and luciferase reporter plasmid containing wild-type (WT) and *miR-145-5p* binding site mutated (Mut) *circPARD3.* N.S., no significant. **f** FD-LSC-1 and Tu 177 cells overexpressing *circPARD3* (circPARD3-OE) were transfected with *miR-145-5p* mimics or NC mimics, expression of LC3B and p62 was detected by western blotting. **g** and **h** Cell proliferation of FD-LSC-1 and Tu 177 cells overexpressing *circPARD3* and miR-145-5p was determined by CCK8 assays (**g**) and colony formation assays (**h**). The migration (**i**) and invasion (**j**) abilities of FD-LSC-1 and Tu 177 cells overexpressing *circPARD3* and transfected with miR-145-5p mimics were evaluated by Transwell assays. Scale bar, 200 μm. **k** LSCC cells overexpressing *circPARD3* and miR-145-5p mimics were treated with various concentrations of Cisplatin for 24 h. Cell viability was determined by CCK8 assays. Error bars represent SD of three independent experiments. *P < 0.05, **P < 0.01

The function of circRNA as miRNA sponge requires it to bind with AGO2 and miRNA to form a circRNA-AGO2-miRNA complex [24]. RNA immunoprecipitation (RIP) experiments confirmed that *circPARD3* bound to AGO2 (Fig. 6a), suggesting its role as a miRNA sponge. Bioinformatics analysis predicted that 54 miRNAs were potential targets of *circPARD3* (Additional file 1: Table S10). Venn analysis of these 54 miRNAs and miRNAs downregulated in the 107 LSCC tissues obtained seven miRNA candidates, including *miR-1298-5p*, *miR-136-5p*, *miR-139-5p*, *miR-145-5p*, *miR-338-3p*, *miR-100-5p*, and *miR-99a-5p* (Fig. 6b). Next, Biotin-labeled probes that specifically target *circPARD3* were used for RNA pulldown, then qPCR was used to detect these seven miRNAs. Results showed that *miR-145-5p* was the highest enriched one among the 7 miRNAs (Fig. 6c). Furthermore, we found that only *miR-145-5p* expression was significantly decreased after *circPARD3* overexpression, whereas *circPARD3* knockdown led to increased *miR-145-5p* expression in both FD-LSC-1 and Tu 177 cells (Fig. 6d; Additional file 3: Figure S5A and B). We constructed *circPARD3* luciferase reporter plasmids with wild-type (circPARD3-WT) or mutant (circPARD3-Mut) *miR-145-5p* binding sites. We observed that the luciferase activity was markedly decreased in cells cotransfected with *miR-145-5p* mimics and circPARD3-WT, but not with circPARD3-Mut (Fig. 6e). These data suggested that *circPARD3* serves as a sponge for *miR-145-5p*.

Next, we investigated whether *miR-145-5p* affected autophagy levels in LSCC cells. Western blotting showed increased LC3-II level and decreased p62 level in FD-LSC-1 and Tu 177 cells after transfection with *miR-145-5p* mimics (Additional file 3: Figure S6A). Conversely, LC3-II was decreased, and p62 was increased after *miR-145-5p* inhibitor transfection (Additional file 3: Figure S6B). Autophagic flux analysis showed the numbers of green and red LC3 puncta increased in the cells transfected with *miR-145-5p* mimics but decreased in those transfected with *miR-145-5p* inhibitor (Additional file 3: Figure S6C), indicating that *miR-145-5p* enhances the autophagy levels in LSCC cells.

Since *miR-145-5p* acts as a tumor suppressor to inhibit LSCC cell proliferation, migration and invasion [25], we wonder whether *circPARD3* inhibits autophagy and promotes malignant progression of LSCC by antagonizing *miR-145-5p*. Western blotting and autophagy flux analysis showed that overexpression of *circPARD3* reversed the activation effect of the *miR-145-5p* mimics on autophagy (Fig. 6f; Additional file 3: Figure S7). CCK8 and colony formation assays revealed that overexpression of *circPARD3* reversed the inhibitory effect of *miR-145-5p* mimics on cell proliferation (Fig. 6g and h). Transwell analysis showed that overexpression of *circPARD3* rescued the decreased migration and invasion caused by *miR-145-5p* mimics transfection (Fig. 6i and j). Moreover, *circPARD3* overexpression also antagonized the chemosensitization effect of *miR-145-5p* mimics on FD-LSC-1 and Tu 177 cells (Fig. 6k). These data suggested that *circPARD3* inhibits autophagy by sponging the tumor suppressor *miR-145-5p*, thereby promoting LSCC cell proliferation, migration, invasion, and chemoresistance.

### PRKCI is a direct target gene of *miR-145-5p*, which functions as a driver gene in LSCC

**Fig. 7:**
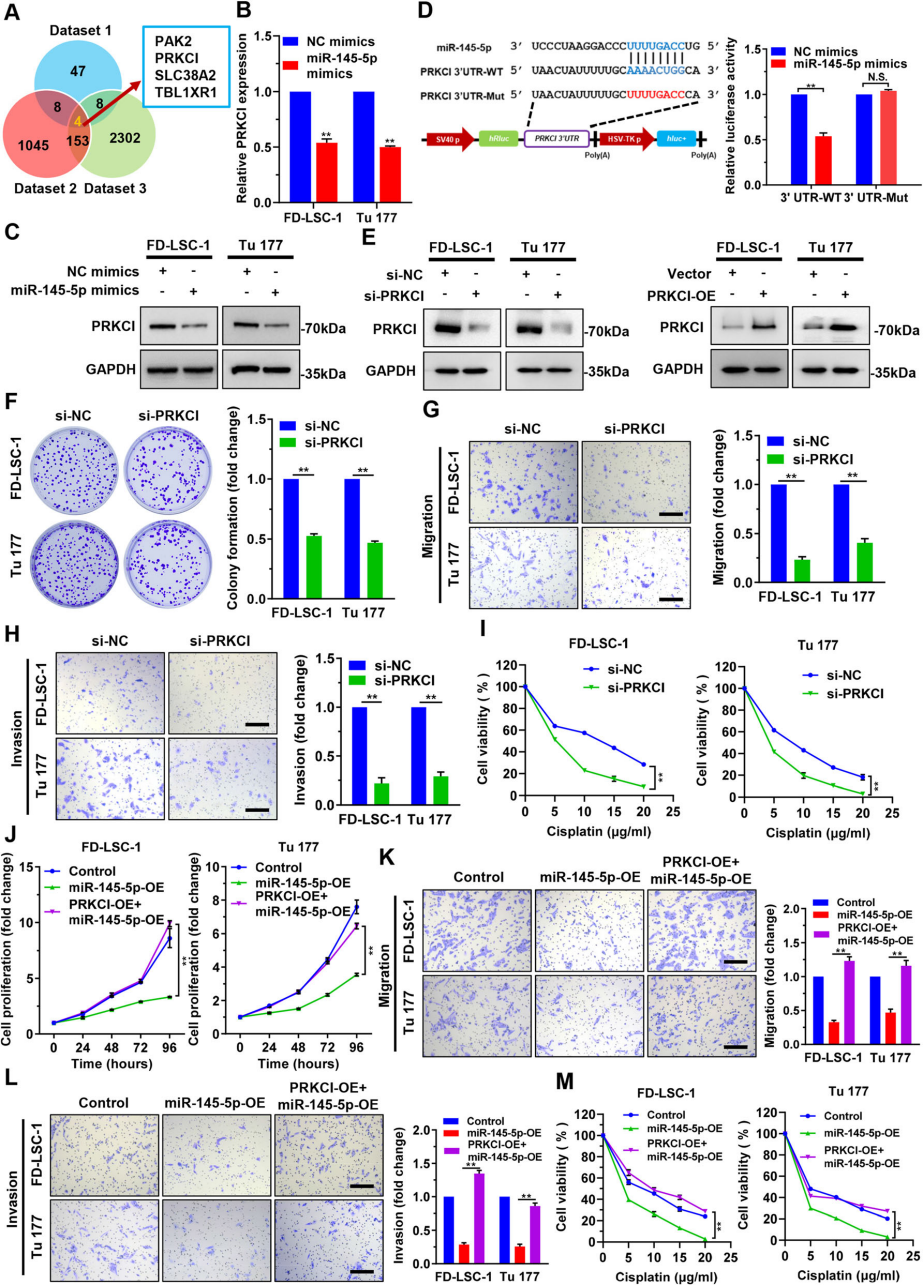
PRKCI is a direct downstream target gene of miR-145-5p in LSCC cells. **a** Venn analysis of mRNAs downregulated in Tu 177 cells overexpressing *miR-145-5p* (Dataset 1), bioinformatics predicted *miR-145-5p* target genes (Dataset 2), and mRNAs upregulated in 107 LSCC tissues from RNA sequencing data (Dataset 3). **b** and **c** FD-LSC-1 and Tu 177 cells were transfected with *miR-145-5p* mimics or NC mimics for 48 h, then the expression of PRKCI was detected by qPCR analysis (**b**) and western blotting (**c**). **d** Luciferase reporter assay of FD-LSC-1 cells cotransfected with *miR-145-5p* mimics and luciferase reporter plasmid containing wild-type (WT) and *miR-145-5p* binding site mutated (Mut) *PRKCI* 3′ UTR sequence. N.S., no significant. **e** Expression of PRKCI in FD-LSC-1 and Tu 177 cells silencing or overexpression of PRKCI was verified by western blotting. **f** Cell proliferation of LSCC cells silencing of PRKCI was determined by colony formation assays. **g** and **h** The migration (**g**) and invasion (**h**) abilities of FD-LSC-1 and Tu 177 cells after PRKCI knockdown were evaluated by Transwell assays. **i** Cells were transfected with si-PRKCI or si-NC for 24 h, then treated with various concentrations of Cisplatin for 24 h. Cell viability was determined by CCK8 assays. **j** FD-LSC-1 and Tu 177 cells were transfected with *miR-145-5p* mimics, or transfected with *miR-145-5p* mimics and infected with PRKCI overexpression lentiviruses simultaneously. Cell proliferation was determined by CCK8 assays. **k** and **l** The migration (**k**) and invasion (**l**) abilities of FD-LSC-1 and Tu 177 cells overexpressing PRKCI and *miR-145-5p* were detected by Transwell assays. Scale bar, 200 μm. **m** FD-LSC-1 and Tu 177 cells overexpressing PRKCI were transfected with *miR-145-5p* mimics for 24 h, then treated with various concentrations of Cisplatin for 24 h. Cell viability was determined by CCK8 assays. Error bars represent SD of three independent experiments. **P < 0.01

To explore the direct downstream target gene of *miR-145-5p*, we investigated mRNAs that might be regulated by *miR-145-5p* in LSCC cells via microarray analysis. Sixty-seven mRNAs were downregulated in Tu 177 cells with *miR-145-5p* overexpression (Dataset 1, Additional file 1: Table S11). Bioinformatics analysis prediction of *miR-145-5p* target gene obtained 1210 potential target genes (Dataset 2, Additional file 1: Table S12). Venn analysis of Dataset 1, Dataset 2, and mRNAs upregulated in the 107 LSCC tissues compared to their paired ANM tissues from the RNA sequencing data (Dataset 3, Additional file 1: Table S13) obtained four intersection genes, *PAK2*, *PRKCI*, *SLC38A2* and *TBL1XR1* (Fig. 7a). Furthermore, we found that only PRKCI mRNA and protein levels were significantly decreased in LSCC cells transfected with *miR-145-5p* mimics (Fig. 7b and c; Additional file 3: Figure S8). Luciferase reporter assays showed that cotransfection of *miR-145-5p* mimics reduced the luciferase activity of the wild-type *PRKCI* 3′ UTR reporter, but not the one with *miR-145-5p* binding site mutated (Fig. 7d). These data suggested that *PRKCI* is a direct target gene of *miR-145-5p*.

We further tested the effect of PRKCI on LSCC cell proliferation, migration, invasion and chemosensitivity. Transfection of LSCC cells with siRNAs targeting PRKCI (si-PRKCI) or infection with PRKCI overexpression lentiviruses (PRKCI-OE) changed PRKCI levels successfully (Fig. 7e). Colony formation and Transwell assays indicated that knockdown of PRKCI inhibited LSCC cell proliferation, migration and invasion (Fig. 7f-h), while overexpression of PRKCI promoted LSCC cell proliferation, migration and invasion (Additional file 3: Figure S9A-C). Furthermore, knockdown of PRKCI enhanced, but overexpression of PRKCI decreased LSCC sensitivity to chemotherapeutic drug Cisplatin (Fig. 7i; Additional file 3: Figure S9D). Rescue experiments demonstrated that PRKCI overexpression reversed the inhibitory effect of *miR-145-5p* on LSCC cell proliferation, migration, and invasion (Fig. 7j-l), and reduced the sensitivity of cells to Cisplatin (Fig. 7m). These results indicated that *PRKCI*, as a direct downstream target gene of *miR-145-5p*, functions as a driver gene in LSCC to promote cell proliferation, migration, invasion and chemoresistance.

### PRKCI inhibits autophagy by activating the Akt-mTOR signaling axis

**Fig. 8:**
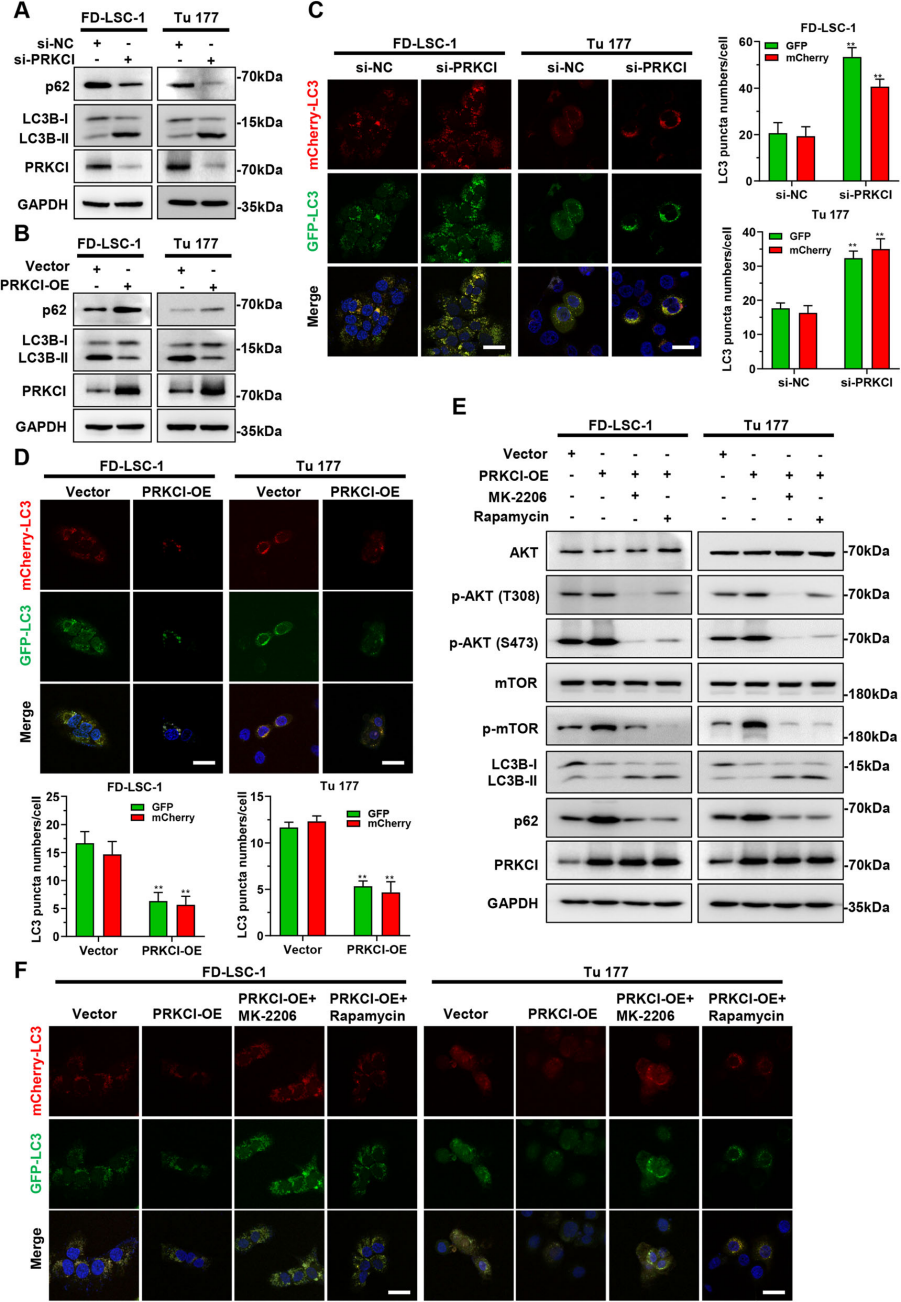
PRKCI inhibits LSCC cell autophagy via activating the Akt-mTOR axis. **a** and **b** The protein levels of PRKCI, LC3B, and p62 in FD-LSC-1 and Tu 177 cells with knockdown (**a**) or overexpression (**b**) of PRKCI were detected by Western blotting. **c** and **d** mCherry-EGFP-LC3B labeled FD-LSC-1 and Tu 177 cells were transfected with si-PRKCI (**c**) or infected with PRKCI overexpression lentiviruses (**d**), autophagic flux was analyzed by confocal microscopy. **e** PRKCI overexpressing FD-LSC-1 and Tu 177 cells (PRKCI-OE) were treated with MK-2206 (10 μM) or Rapamycin (100 nM) for 24 h, the protein levels of phosphorylated Akt (p-AKT(T308), p-AKT(S473)), phosphorylated mTOR (p-mTOR), LC3B, and p62 were detected by western blotting. **f** mCherry-EGFP-LC3B labeled FD-LSC-1 and Tu 177 cells were infected with PRKCI overexpression lentiviruses for 48 h, then treated with MK-2206 (10 μM) or Rapamycin (100 nM) for 24 h. Autophagic flux was analyzed by confocal microscopy. Scale bar, 25 μm. Error bars represent SD of three independent experiments. **P < 0.01

Next, we investigated the effect of PRKCI on autophagy in LSCC cells. FD-LSC-1 and Tu 177 cells with PRKCI knockdown showed higher LC3-II and lower p62 levels than that of control cells (Fig. 8a). By contrast, LC3-II level was decreased, but p62 level was increased after PRKCI overexpression (Fig. 8b). Autophagy flux detection revealed that autophagy level was increased in LSCC cells with PRKCI knockdown and decreased in cells with PRKCI overexpression (Fig. 8c and d), suggesting that PRKCI is an autophagy suppressor in LSCC.

As a member of the protein kinase C family, PRKCI directly phosphorylates Akt and activates the Akt signaling pathway, and the activated Akt pathway could suppress autophagy by phosphorylating mTOR [26]. To examine whether PRKCI inhibits autophagy in LSCC cells by activating the Akt-mTOR signaling axis, PRKCI overexpressing LSCC cells were treated with Akt phosphorylation inhibitor MK-2206 or mTOR phosphorylation inhibitor Rapamycin. We observed that PRKCI overexpression enhances protein phosphorylation levels of Akt and mTOR (Fig. 8e). However, MK-2206 and Rapamycin treatment eliminated the increased phosphorylation of Akt and mTOR induced by PRKCI (Fig. 8e). Furthermore, we found that MK-2206 and Rapamycin treatment reversed the inhibitory effect of PRKCI on FD-LSC-1 and Tu 177 cell autophagy (Fig. 8e and f). These data confirmed that PRKCI inhibits LSCC cell autophagy by activating the Akt-mTOR signaling axis.

### *circPARD3* inhibits LSCC cell autophagy via the PRKCI-Akt-mTOR pathway

**Fig. 9:**
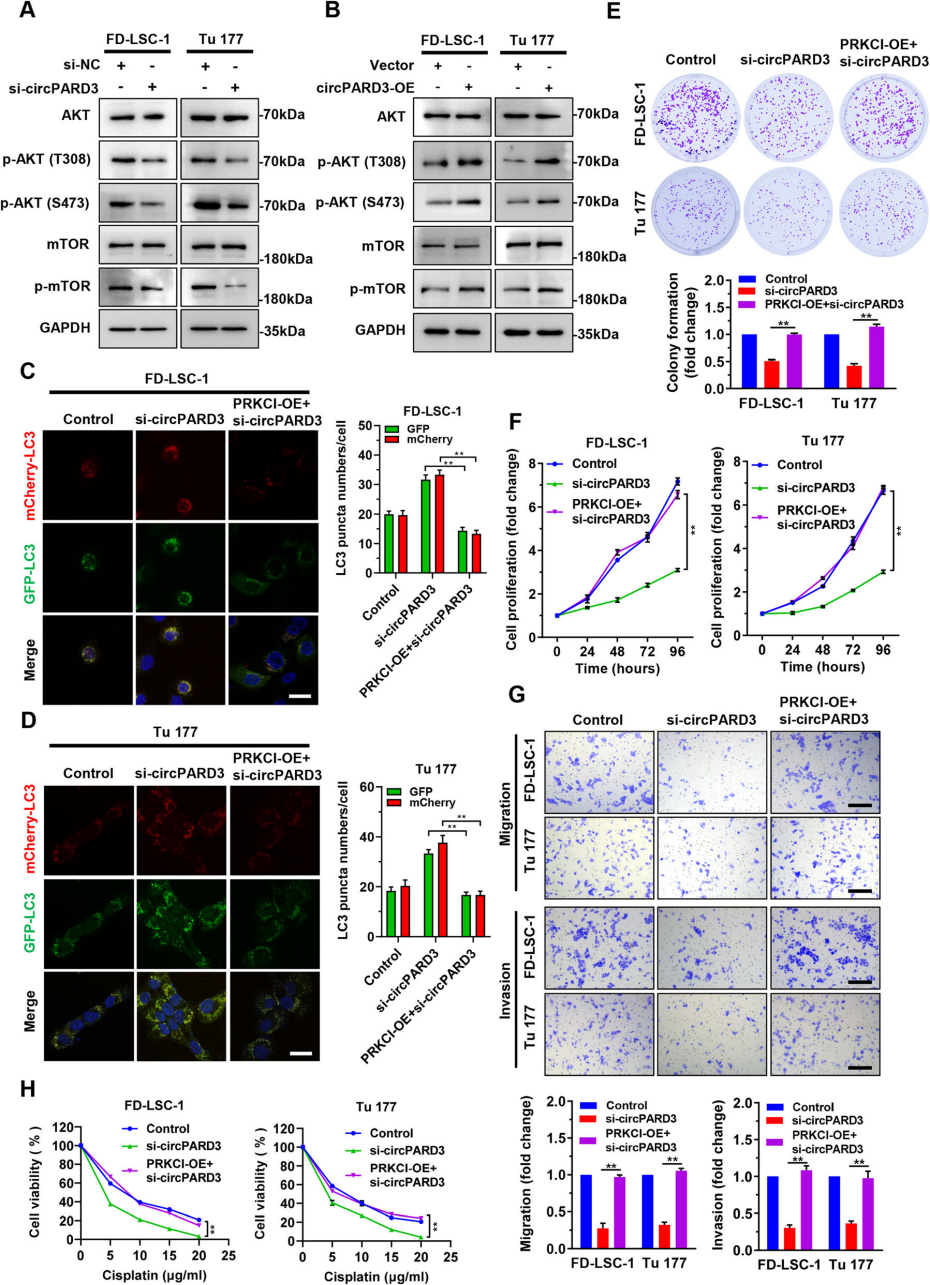
Overexpression of PRKCI reverses phenotype changes induced by *circPARD3* knockdown. **a** and **b** The protein levels of phosphorylated Akt (p-AKT(T308), p-AKT(S473)), and phosphorylated mTOR (p-mTOR) in FD-LSC-1 and Tu 177 cells with knockdown (**a**) or overexpression (**b**) of *circPARD3* were determined by western blotting. **c** and **d** mCherry-EGFP-LC3B labeled FD-LSC-1 (**c**) and Tu 177 (**d**) cells were transfected with si-circPARD3 alone or transfected with si-circPARD3 and infected with PRKCI overexpression lentiviruses for 48 h, autophagic flux was analyzed by confocal microscopy. Scale bar, 25 μm. **e** and **f** FD-LSC-1 and Tu 177 cells were transfected with si-circPARD3 alone or transfected with si-circPARD3 and infected with PRKCI overexpression lentiviruses, cell proliferation was evaluated by colony formation assays (**e**) and CCK8 analysis (**f**). **g** The migration and invasion abilities of FD-LSC-1 and Tu 177 cells with circPARD3 knockdown alone or circPARD3 knockdown and PRKCI overexpression were detected by Transwell assays. Scale bar, 200 μm. **h** FD-LSC-1 and Tu 177 cells were transfected with si-circPARD3 alone or transfected with si-circPARD3 and infected with PRKCI overexpression lentiviruses for 48 h, then treated with various concentrations of Cisplatin for 24 h. Cell viability was determined by CCK8 assays. Error bars represent SD of three independent experiments. **P < 0.01

Subsequently, we further assessed the effects of *circPARD3* on the PRKCI-Akt-mTOR pathway. Western blotting revealed that *circPARD3* knockdown decreased protein phosphorylation levels of Akt and mTOR (Fig. 9a), while *circPARD3* overexpression led to increased phosphorylation of Akt and mTOR (Fig. 9b), suggesting that *circPARD3* is able to activate the PRKCI-Akt-mTOR pathway. Autophagy flux analysis showed that PRKCI overexpression attenuated the increased autophagy induced by *circPARD3* knockdown (Fig. 9c and d). Furthermore, we found that PRKCI overexpression rescued the decreased cell proliferation, migration, invasion and chemoresistance abilities caused by *circPARD3* knockdown (Fig. 9e–h).

**Fig. 10:**
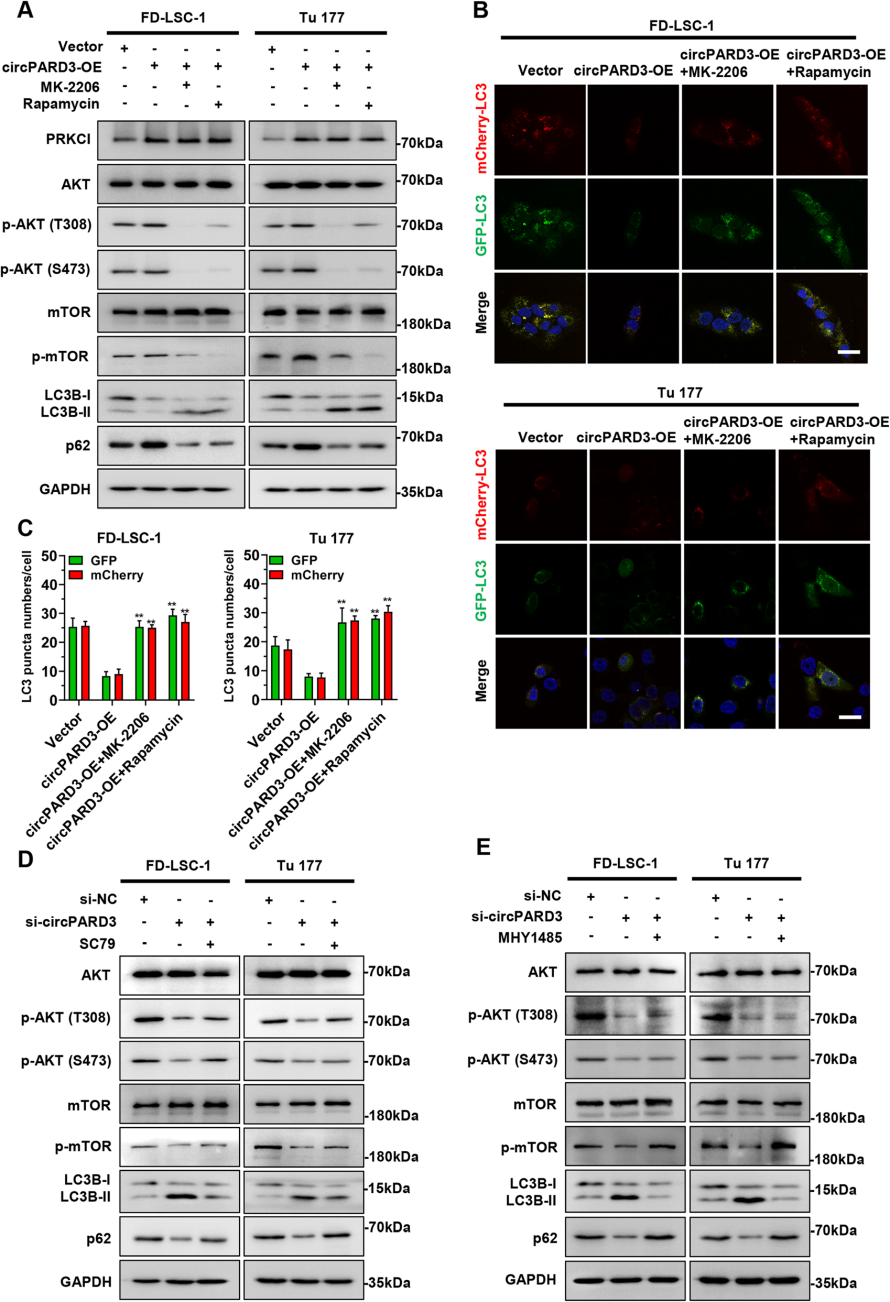
*circPARD3* inhibits autophagy of LSCC cells via the PRKCI-Akt-mTOR pathway. **a** FD-LSC-1 and Tu 177 cells with *circPARD3* overexpression (circPARD3-OE) were treated with MK-2206 (10 μM) or Rapamycin (100 nM) for 24 h, then the protein levels of phosphorylated Akt (p-AKT(T308), p-AKT(S473)), phosphorylated mTOR (p-mTOR), LC3B, and p62 were detected by western blotting. **b** and **c** mCherry-EGFP-LC3B labeled FD-LSC-1 and Tu 177 cells were infected with *circPARD3* overexpression lentiviruses for 48 h, then treated with MK-2206 (10 μM) or Rapamycin (100 nM) for 24 h. Autophagic flux was analyzed by confocal microscopy. Representative confocal microscopy images (**b**) and quantitative data (**c**) were shown. Scale bar, 25 μm. **d** and **e** FD-LSC-1 and Tu 177 cells were transfected with si-circPARD3 for 24 h, then cells were treated with Akt activator SC79 (5 μg/ml) (**d**) or mTOR activator MHY1485 (10 μM) (**e**) for 24 h. The protein levels of phosphorylated Akt (p-AKT(T308), p-AKT(S473)), phosphorylated mTOR (p-mTOR), LC3B, and p62 were detected by western blotting. Error bars represent SD of three independent experiments. **P < 0.01

To validate the significance of the PRKCI-Akt-mTOR pathway for *circPARD3*-mediated autophagy inhibition, *circPARD3* overexpressing LSCC cells were treated with MK-2206 or Rapamycin, respectively. Western blotting and autophagy flux analysis showed that inhibition of Akt or mTOR phosphorylation reactivated autophagy inhibited by *circPARD3* overexpression (Fig. 10a-c). We further compared the changes of autophagy levels in *circPARD3* knockdown cells treated with the Akt activator SC79 or the mTOR activator MHY1485. Expectedly, autophagy levels in SC79 or MHY1485 treatment cells were lower than that of nontreatment controls (Fig. 10d and e; Additional file 3: Figure S10). Collectively, these results suggested that the PRKCI-Akt-mTOR signaling pathway is the main pathway mediating the inhibitory effect of *circPARD3* on autophagy in LSCC cells.

### Silencing of *circPARD3* inhibits tumorigenicity and enhances chemosensitivity of LSCC cell in preclinical models

**Fig. 11:**
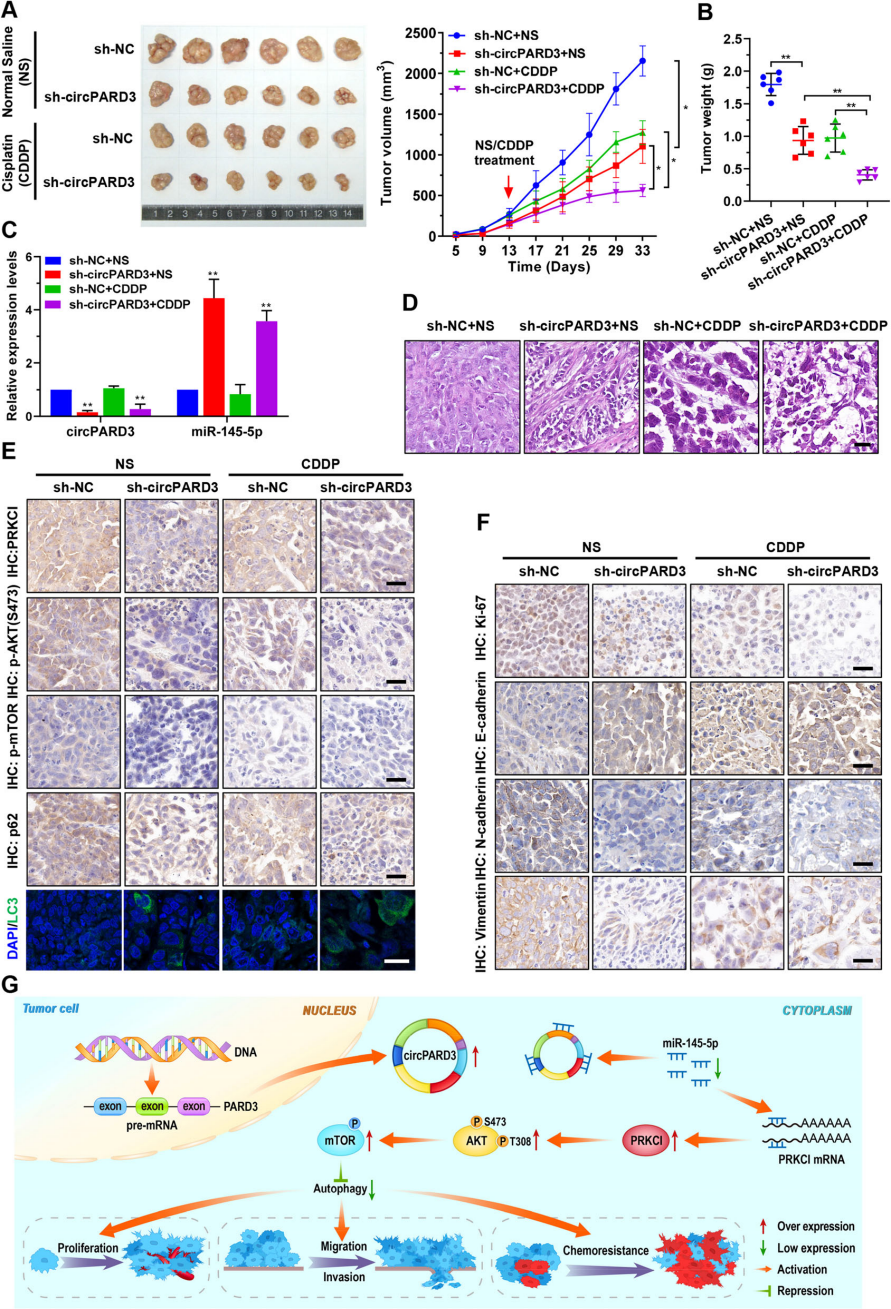
Silencing of *circPARD3* suppresses tumorigenicity and enhances chemosensitivity of LSCC cells in vivo. **a** Effects of *circPARD3* on LSCC xenograft tumor growth and chemosensitivity in vivo. Left: Representative images of tumors in nude mice after subcutaneous injection of FD-LSC-1 cells with *circPARD3* knockdown (sh-circPARD3) and treated with Cisplatin (*n* = 6). Right: Tumor growth curve was plotted using xenograft tumor volume data. **b** Tumor weight was measured after tumor excision. **c** The relative expression levels of *circPARD3* and *miR-145-5p* in xenograft tumors were determined by qPCR analysis. **d** Hematoxylin and eosin staining revealed the structure of xenograft tumors. Scale bar, 20 μm. **e** IHC staining of PRKCI, p-AKT(S473), p-mTOR, p62 and immunofluorescence of LC3B in xenograft tumors. Scale bar, 25 μm. **f** IHC staining of Ki-67, E-cadherin, N-cadherin, and Vimentin in xenograft tumors. Scale bar, 25 μm. **g** Schematic illustration indicates the mechanism by which *circPARD3* inhibits autophagy to promote malignant progression and chemoresistance of LSCC via activating the PRKCI–Akt–mTOR pathway. NS, Normal Saline. Data are mean ± SD. *P < 0.05, **P < 0.01

To further investigate the functional role of *circPARD3* in LSCC in vivo, we constructed FD-LSC-1 cells with stably knockdown of *circPARD3* (sh-circPARD3) and control cells (sh-NC), and established xenograft tumor models in BALB/c nude mice. The tumor-bearing mice were treated with Cisplatin or normal saline for 3 weeks (the experimental group was intraperitoneally injected with 1 mg/kg of Cisplatin every 2 days; the control group was injected with the equal volume of normal saline). Compared with the control group, xenograft tumor growth rate was slower, and tumor weight was significantly lower in the sh-circPARD3 group (Fig. 11a and b). Notably, the sh-circPARD3 group treated with Cisplatin showed the slowest growth rate among all four groups (Fig. 11a and b). qPCR analysis demonstrated that *circPARD3* was decreased, but *miR-145-5p* was increased in the sh-circPARD3 groups when compared with the sh-NC groups (Fig. 11c). Hematoxylin and eosin staining showed that the tumor cells in sh-circPARD3 knockdown group of xenograft tumors was significantly reduced (Fig. 11d).

IHC staining revealed that the expression levels of PRKCI, phosphorylated Akt, phosphorylated mTOR, and p62 were decreased obviously in the tumors collected from sh-circPARD3 groups compared with those from the sh-NC groups (Fig. 11e). Immunofluorescence showed increased LC3 dots in xenograft tumors collected from sh-circPARD3 groups (Fig. 11e). Furthermore, the proliferation marker Ki-67 and the EMT markers N-cadherin, and Vimentin were decreased in xenograft tumors with *circPARD3* knockdown (Fig. 11f). In contrast, E-cadherin level was increased in xenograft tumors with *circPARD3* knockdown (Fig. 11f). These results demonstrated that silencing of *circPARD3* inhibited tumorigenicity and enhanced chemosensitivity of LSCC cells in vivo by enhancing autophagy through the PRKCI-Akt-mTOR pathway.

## Discussion

LSCC is characterized by local recurrences, lymph node metastasis and poor sensitivity to chemotherapeutic drugs, which severely restricts the effects of LSCC treatment [2, 27]. Autophagy plays critical roles in regulation of invasion, metastasis, and chemosensitivity in various type of cancers [28–32]. Therefore, scientists and clinicians are attempt to explore new cancer treatment approach by modulating autophagy. In this study, we found that the autophagy substrate protein p62 was significantly upregulated in LSCC tissues, and high level of p62 was associated with malignant progression and poor prognosis of LSCC patients, indicating that autophagy inhibition is an important molecular event in LSCC initiation and development. Moreover, these data also suggested that the anti-tumor effect of autophagy is continuously inhibited during LSCC initiation and progression. Therefore, reactivation of autophagy may be able to suppress LSCC cell malignant behaviors. Remarkably, our data demonstrated that upregulation of autophagy level inhibited proliferation, migration, invasion, and enhanced chemosensitivity of LSCC cells to Cisplatin in vitro and in vivo. Similarly, Zhu et al. reported that upregulation of autophagy level inhibited nasopharyngeal carcinoma cell invasion and metastasis [33], confirming that activation of autophagy might be a new strategy for LSCC treatment.

We further screened the autophagy-related circRNAs by RNA sequencing of LSCC tissues and autophagic flux analysis, and identified a novel autophagy-suppressive circRNA *circPARD3*. *circPARD3* was abnormally upregulated in LSCC tissues compared with their paired normal tissues. Importantly, we found that high *circPARD3* level was closely associated with malignant progression and poor prognosis of LSCC patients. In vivo and in vitro experiments confirmed that *circPARD3* promoted LSCC cell proliferation, migration, invasion and chemoresistance by inhibiting autophagy. To the best of our knowledge, for the first time, this study reveals the intrinsic molecular mechanism of LSCC maintains low autophagy levels via circRNA and clarifies the oncogenic role of *circPARD3* in LSCC. Our findings suggested that *circPARD3* can serve as a biomarker for diagnosis and prognosis of LSCC. More importantly, *circPARD3* can serve as a new biomarker and target for autophagy detection and modulation, which might be used for controlling autophagy levels in different developmental stages of LSCC.

Sponging target miRNAs and inhibiting their functions are classic mechanisms of circRNA. *circRNA-002178* upregulates Programmed death-ligand 1 (PD-L1) expression in lung adenocarcinoma by sponging *miR-34* [34]. *circAMOTL1* enhances expression of the parent gene AMOTL1 by sponging *miR-485-5p*, which promotes cervical cancer progression [35]. Furthermore, *circCDYL* upregulates ATG7 and ULK1 expression by sponging *miR-1275*, thereby enhancing the autophagy levels and malignant progression of breast cancer [36]. We found that *circPARD3* was mainly localized in the cytoplasm and inhibited LSCC cell autophagy by sponging *miR-145-5p*. Previous studies reported that *miR-145-5p* promotes autophagy of cardiomyocytes and human umbilical cord-derived mesenchymal stem cells by inhibiting the Akt signaling pathway [37, 38], supporting our conclusion that *miR-145-5p* promotes autophagy in LSCC cells.

As a member of the protein kinase C family, PRKCI affects tumorigenesis, progression and chemosensitivity by regulating the Wnt/beta-catenin pathway and immune microenvironment in lung cancer, ovarian cancer and alveolar rhabdomyosarcoma [39–41]. Therefore, PRKCI is considered an important target for cancer treatment. Our RNA sequencing data showed that *PRKCI* was upregulated in LSCC, and we demonstrated that PRKCI was a new target gene of *miR-145-5p*. Moreover, functional studies demonstrated that PRKCI inhibited autophagy and promoted LSCC cell proliferation, migration, invasion and chemoresistance. Qu et al. found that knockdown of PRKCI in U2OS cells resulted in elevated autophagy levels and inhibiting the malignant cell phenotype [26]. Consistently, our study revealed that PRKCI enhanced Akt and mTOR phosphorylation and inhibited LSCC cell autophagy. Furthermore, blocking Akt and mTOR activity using small-molecule inhibitors eliminated the inhibitory effect of PRKCI on autophagy, indicating that PRKCI inhibits autophagy by activating the Akt-mTOR pathway in LSCC cells. In addition, our data demonstrated that *circPARD3* inhibited autophagy and promoted malignant progression and chemoresistance of LSCC cells via activating the PRKCI-Akt-mTOR pathway. Taken together, our study linked *circPARD3* with autophagy, LSCC progression and chemoresistance.

Finally, our study revealed that knockdown of *circPARD3* inhibited the tumorigenicity and enhanced chemosensitivity of LSCC cells in preclinical models. Furthermore, IHC staining results confirmed that downregulation of *circPARD3* inhibited the PRKCI-Akt-mTOR pathway and activated autophagy in vivo. These results indicated that activating autophagy is a potential strategy for LSCC treatment, and developing techniques for *circPARD3* detection and intervention to restore autophagy activity have great potential for LSCC diagnosis and treatment.

## Conclusion

In summary, we demonstrated that autophagy is inhibited in LSCC, and that *circPARD3* inhibits autophagy to promote malignant and chemoresistance of LSCC. Our study further revealed the mechanism of *circPARD3* regulates autophagy via the miR-145-5p-PRKCI-Akt-mTOR pathway (Fig. 11g). Collectively, these findings provide new insights into the role and mechanism of circRNAs in autophagy, progression, and chemoresistance of LSCC. Moreover, the current study highlights the importance of restoring autophagy in LSCC treatment, and demonstrates the potential of *circPARD3* as a biomarker and effective target for LSCC diagnosis and treatment.

## Supplementary Information

**Additional file 1: Table S1.** Clinicopathological characteristics of 138 LSCC samples (cohort 1) for IHC staining of p62. **Table S2.** Clinicopathological characteristics of 107 LSCC samples (cohort 2) for RNA sequencing. **Table S3.** Clinicopathological characteristics of 100 LSCC samples (cohort 3) for qPCR analysis of circPARD3. **Table S4.** Detailed information of inhibitors. **Table S5.** Cell lines and growth medium**. Table S6.** Primer information for qPCR analysis. **Table S7.** Differentially expressed circRNAs in RNA sequencing data of 107 LSCC and paired ANM tissues. **Table S8.** Differentially expressed miRNAs in RNA sequencing data of 107 LSCC and paired ANM tissues. **Table S9.** Differentially expressed mRNAs in RNA sequencing data of 107 LSCC and paired ANM tissues. **Table S10.** Prediction of *circPARD3* binding miRNAs by CircInteractome. **Table S11.** Downregulated genes in Tu 177 cells transfected with *miR-145-5p* mimics analyzed by microarray. **Table S12.** Prediction of *miR-145-5p* target gene by Targetminer. **Table S13.** Upregulated mRNAs in RNA sequencing data of 107 LSCC and paired ANM tissues

**Additional file 2.** Supplemental Materials and Methods.

**Additional file 3: Figure S1.** The flow chart for screening and verifying autophagy suppressive *circPARD3* in LSCC. **Figure S2.** FD-LSC-1 and Tu 177 cells were transfected with Cy3 labeled si-NC (si-NC-Cy3), NC mimics (NC mimics-Cy3), or NC inhibitor (NC inhibitor-Cy3) for 48 h. Nuclei were stained with DAPI (blue). Transfection efficiency was evaluated by imaging with confocal microscopy. Red dot represents siRNA, miRNA mimics, or miRNA inhibitor. Scale bar, 50 μm. **Figure S3.** Verification of the structure features of *circPARD3*. **a** Expression of *circPARD3* in FD-LSC-1 and Tu 177 cells was verified by RT-PCR. Agarose gel electrophoresis showed that divergent primers amplified *circPARD3* in cDNA but not genomic DNA (gDNA). GAPDH served as a negative control. **b** Stability of *circPARD3* and linear *PARD3* mRNA was assessed by RNase R treatment and RT-PCR analysis. **Figure S4**. FD-LSC-1 and Tu 177 cells were infected with *circPARD3* overexpression lentiviruses (circPARD3-OE) or transfected with si-*circPARD3* (si-circ-1, si-circ-2) for 48 h. Expression level of linear *PARD3* mRNA was determined by qPCR analysis. Error bars represent SD of three independent experiments. N.S., no significant. **Figure S5.** Expression levels of potential *circPARD3* target miRNAs in FD-LSC-1 and Tu 177 cells with overexpression (**a**) or knockdown (**b**) of *circPARD3* were determined by qPCR analysis. Error bars represent SD of three independent experiments. * *P* < 0.05, **P < 0.01. **Figure S6.** The effects of *miR-145-5p* on LSCC cell autophagy. **a** and **b** FD-LSC-1 and Tu 177 cells were transfected with *miR-145-5p* mimics (**a**) or inhibitor (**b**) for 48 h. Expression levels of p62 and LC3B were detected by western blotting. **c** FD-LSC-1 and Tu 177 cells were transfected with *miR-145-5p* mimics or inhibitor for 48 h. Autophagic flux was analyzed by confocal microscopy. Representative images (Top) and statistical data (Bottom) were shown. Scale bar, 25 μm. Error bars represent SD of three independent experiments. * *P* < 0.05, **P < 0.01. **Figure S7.** mCherry-EGFP-LC3B labeled FD-LSC-1 and Tu 177 cells were transfected with miR-145-5p mimics alone or infected with *circPARD3* overexpression lentiviruses simultaneously for 48 h*.* Autophagic flux was analyzed by confocal microscopy. Representative images (Top) and statistical data (Bottom) were shown. Scale bar, 25 μm. Error bars represent SD of three independent experiments. * *P* < 0.05, **P < 0.01. **Figure S8.** FD-LSC-1 and Tu 177 cells were transfected with miR-145-5p mimics or NC mimics for 48 h, then expression levels of *PAK2*, *SLC38A2*, and *TBL1XR1* were determined by qPCR analysis. Error bars represent SD of three independent experiments. N.S., no significant. **Figure S9.** The effects of PRKCI overexpression on LSCC cell proliferation, migration, invasion, and chemoresistance. **a** Cell proliferation of FD-LSC-1 and Tu 177 cells overexpressing PRKCI was determined by colony formation assays. **b** and **c** The migration (**b**) and invasion (**c**) abilities of FD-LSC-1 and Tu 177 cells overexpressing PRKCI were evaluated by Transwell assays. Scale bar, 200 μm. **d** FD-LSC-1 and Tu 177 cells overexpressing PRKCI were treated with various concentrations of Cisplatin for 24 h. Cell viability was determined by CCK8 assays. Error bars represent SD of three independent experiments. **P < 0.01. **Figure S10.** FD-LSC-1 and Tu 177 cells were transfected with si-circPARD3 for 24 h, then treated with Akt activator SC79 (5 μg/ml) or mTOR activator MHY1485 (10 μM) for 24 h. Autophagic flux was analyzed by confocal microscopy. Representative images (Left) and statistical data (Right) were shown. Scale bar, 25 μm. Error bars represent SD of three independent experiments. **P < 0.01.

## Data Availability

RNA sequencing raw data and normalized results were deposited at GEO database (GSE132222, GSE130605, GSE127165, and GSE133632). All data that support the findings of this study are available from the corresponding authors upon reasonable request.

## Abbreviations

LSCC: Laryngeal squamous cell carcinoma
ANM: Adjacent normal mucosa
circRNA: Circular RNA
EMT: Epithelial–mesenchymal transition
qPCR: Quantitative real-time PCR
FISH: Fluorescence in situ hybridization
RIP: RNA immunoprecipitation
3′ UTR: 3′-untranslated region
mTOR: Mammalian target of rapamycin
DAPI: 4′,6-diamidino-2-phenylindole
