# Supplementary material for: circPARD3 drives malignant progression and chemoresistance of laryngeal squamous cell carcinoma by inhibiting autophagy through the PRKCI-Akt-mTOR pathway
Source: Mol Cancer. 2020 Nov 24;19:166. doi: 10.1186/s12943-020-01279-2 (PMC7686732; doi:10.1186/s12943-020-01279-2)
Supplement: Supplementary file 1 — Additional file 1: Table S1. Clinicopathological characteristics of 138 LSCC samples (cohort 1) for IHC staining of p62. Table S2. Clinicopathological characteristics of 107 LSCC samples (cohort 2) for RNA sequencing. Table S3. Clinicopathological characteristics of 100 LSCC samples (cohort 3) for qPCR analysis of circPARD3. Table S4. Detailed information of inhibitors. Table S5. Cell lines and growth medium. Table S6. Primer information for qPCR analysis. Table S7. Differentially expressed circRNAs in RNA sequencing data of 107 LSCC and paired ANM tissues. Table S8. Differentially expressed miRNAs in RNA sequencing data of 107 LSCC and paired ANM tissues. Table S9. Differentially expressed mRNAs in RNA sequencing data of 107 LSCC and paired ANM tissues. Table S10. Prediction of circPARD3 binding miRNAs by CircInteractome. Table S11. Downregulated genes in Tu 177 cells transfected with miR-145-5p mimics analyzed by microarray. Table S12. Prediction of miR-145-5p target gene by Targetminer. Table S13. Upregulated mRNAs in RNA sequencing data of 107 LSCC and paired ANM tissues [file 12943_2020_1279_MOESM1_ESM.zip › Table S5. Cell lines and culture media.docx]

| **Cell line** | **Complete growth medium** |
| --- | --- |
| HEK293T | DMEM (Gibco: Cat# 10566024) + 10% FBS (Biological Industries, Cat# 04-001-1ACS) |
| MRC-5 | MEM (Gibco: Cat# 11095098) + 10% FBS (Biological Industries, Cat# 04-001-1ACS) |
| FD-LSC-1 | BEGM (Lonza: Cat# CC-3170) + 10% FBS (Biological Industries, Cat# 04-001-1ACS) |
| Tu 177 | DMEM (Gibco: Cat# 10566024) + 10% FBS (Biological Industries, Cat# 04-001-1ACS) |
| HOK | DMEM (Gibco: Cat# 10566024) + 10% FBS (Biological Industries, Cat# 04-001-1ACS) |

**Table S5. Cell lines and growth medium.**
