# Supplementary material for: circPARD3 drives malignant progression and chemoresistance of laryngeal squamous cell carcinoma by inhibiting autophagy through the PRKCI-Akt-mTOR pathway
Source: Mol Cancer. 2020 Nov 24;19:166. doi: 10.1186/s12943-020-01279-2 (PMC7686732; doi:10.1186/s12943-020-01279-2)
Supplement: Supplementary file 2 — Additional file 2. Supplemental Materials and Methods. [file 12943_2020_1279_MOESM2_ESM.docx]

**Supplemental Materials and Methods**

**Agarose gel electrophoresis**

PCR products were separated by 2% agarose gel electrophoresis with TAE buffer using a 100 bp DNA ladder (TransGen Biotech). The bands were photographed under an Azure C600 imager (Azure Biosystems, Dublin, CA).

**Fluorescence *in situ* hybridization (FISH)**

Cy3-labeled *circPARD3* probes (5’- ACTGTAGGGGACAGCTGGTATTTACCTGTCTACAGGTGAG-3’) were synthesized by Sangon Biotech (Shanghai, China). A FISH kit (RiboBio, Guangzhou, China) was used to detect probe signals following the manufacturer’s instructions. Nuclei were stained with DAPI. Images were acquired on a Leica TCS SP8 confocal laser scanning microscope (Leica Microsystems Inc., Buffalo Grove, IL).

**Transient and stable transfection of cells**

Transient transfection of miRNA mimics, inhibitor, and siRNAs was performed using Lipofectamine 3000 (Invitrogen) according to the manufacturer’s instructions. For stable transfections, lentiviruses were generated through cotransfection of HEK293T cells with lentiviral plasmids and packaging plasmids GAG and VSVG using Lipofectamine 3000. Virus supernatant was harvested 48 h after transfection, mixed with polybrene (8 μg/ml), and added to target cells (FD-LSC-1, Tu 177). After 48 h incubation, puromycin (Santa Cruz Biotechnology, Dallas, TX) was added for 1 week to screen stable transfection of cells.

**Cell proliferation and chemosensitivity assays**

Cells were inoculated into a 96-well plate (3×10^3^/well). At 0, 24, 48, 72, and 96 h after seeding, each well was replaced with 100 μL fresh complete medium containing 10 μL TransDetect CCK (TransGen Biotech, Beijing, China) followed by incubation for 1 h. The absorbance of the solution was measured at 450 nm using a Spectra Max i3x Multifunctional microplate detection system (Molecular Devices, San Jose, CA). For chemosensitivity assay, 24 h after inoculation, cells were treated with various concentration of Cisplatin (0, 5, 10, 15, 20 μg/mL) for additional 24 h, then CCK8 assay was performed to determine the cell viability.

**Colony formation assay**

Cells were seeded into 6-well plates at a density of 800 cells/well, and then cultured for 2 weeks. Cells were washed with PBS once and colonies were fixed with 4% paraformaldehyde for 20 min and stained with 0.1% crystal violet solution for 10 min at room temperature, followed by image capture.

**Transwell migration and invasion assays**

The migration ability of FD-LSC-1 and Tu 177 cells was assessed using 24-well Transwell system (Corning, Tewksbury, MA) with 8 μm pore size of PET filter membrane. Cells were digested, washed twice with PBS and resuspended in serum-free medium. Transwell chambers for invasion assay were precoated with Matrigel (BD Biosciences, San Jose, CA). 200 μL Serum-free medium containing cells (1×10^5^ cells for invasion assay, 4×10^4^ cells for migration assay) was seeded into the top chamber. The lower chamber contained medium supplemented with 20% FBS. After 24 h, cells in the top chamber were removed and the lower chamber was gently washed with PBS and fixed with 4% paraformaldehyde, stained with 0.1% crystal violet, then images were captured by microscope.

**RNA immunoprecipitation (RIP) assay**

RIP assays were performed with a Magna RIP RNA-Binding Protein Immunoprecipitation Kit (Millipore, Billerica, MA) according to the manufacturer’s instructions. Briefly, 1×10^7^ cells were collected and resuspended in 300 μL RIP lysis buffer. Five micrograms of AGO2 antibody (#2897; CST, Danvers, MA) or normal rabbit IgG was incubated with the cell lysate and protein A/G magnetic beads at 4°C overnight with rotation. Immunoprecipitated RNA was purified and qPCR was performed to evaluate the enrichment of *circPARD3*.

**Luciferase Reporter Assay**

Luciferase reporter assay was conducted in FD-LSC-1 cells. Cells were cotransfected with luciferase reporter plasmid and *miR-145-5p* mimics or NC mimics for 48 h. The luciferase activity was measured using a dual luciferase reporter assay system (Promega, Madison, WI) on a Spectra Max i3x Multifunctional microplate detection system (Molecular Devices).

**Immunohistochemical (IHC) staining**

Tissues were fixed in 4% (v/v) formaldehyde in PBS, embedded in paraffin, and cut into 3-μm sections. Sections underwent dewaxing, re-hydration, antigen retrieval, and blocking, and then were incubated with primary antibodies overnight at 4°C in a moist chamber, and washed three times with PBST. Then sections were incubated with HRP-conjugated secondary antibody (CST) for 15 min at room temperature, washed three times with PBST, and then stained with DAB and hematoxylin. Next, sections were dehydrated and mounted with coverslips. The IHC slides were scanned on Panoramic slide scanner II (3D HISTECH, Budapest, Hungary) and quantified with Case Viewer soft version 2.3 (3D HISTECH) to obtain the H-Score.

**Western blotting**

Cells were washed with PBS twice and harvested, then cell lysates were prepared with RIPA buffer containing protease inhibitor cocktail (ThermoFisher Scientific) and centrifuged at 12,000 rpm for 15 min at 4°C. The protein concentration of was determined and equal amounts of total protein were separated by SDS-PAGE and transferred onto PVDF membranes (Millipore, Billerica, MA). The membranes were blocked with 5% non-fat milk in TBST for 2 h at room temperature, then incubated with primary antibodies overnight at 4°C. Then membranes were incubated with HRP-conjugated secondary antibody for 1 h at room temperature. The band signals were visualized with Western Bright ECL HRP substrate (Advansta Inc., San Jose, CA) and captured by a chemiluminescence imaging system (SageCreation Science, Beijing, China).
