# Supplementary figures and images for: circPARD3 drives malignant progression and chemoresistance of laryngeal squamous cell carcinoma by inhibiting autophagy through the PRKCI-Akt-mTOR pathway
Source: Mol Cancer. 2020 Nov 24;19:166. doi: 10.1186/s12943-020-01279-2 (PMC7686732; doi:10.1186/s12943-020-01279-2)

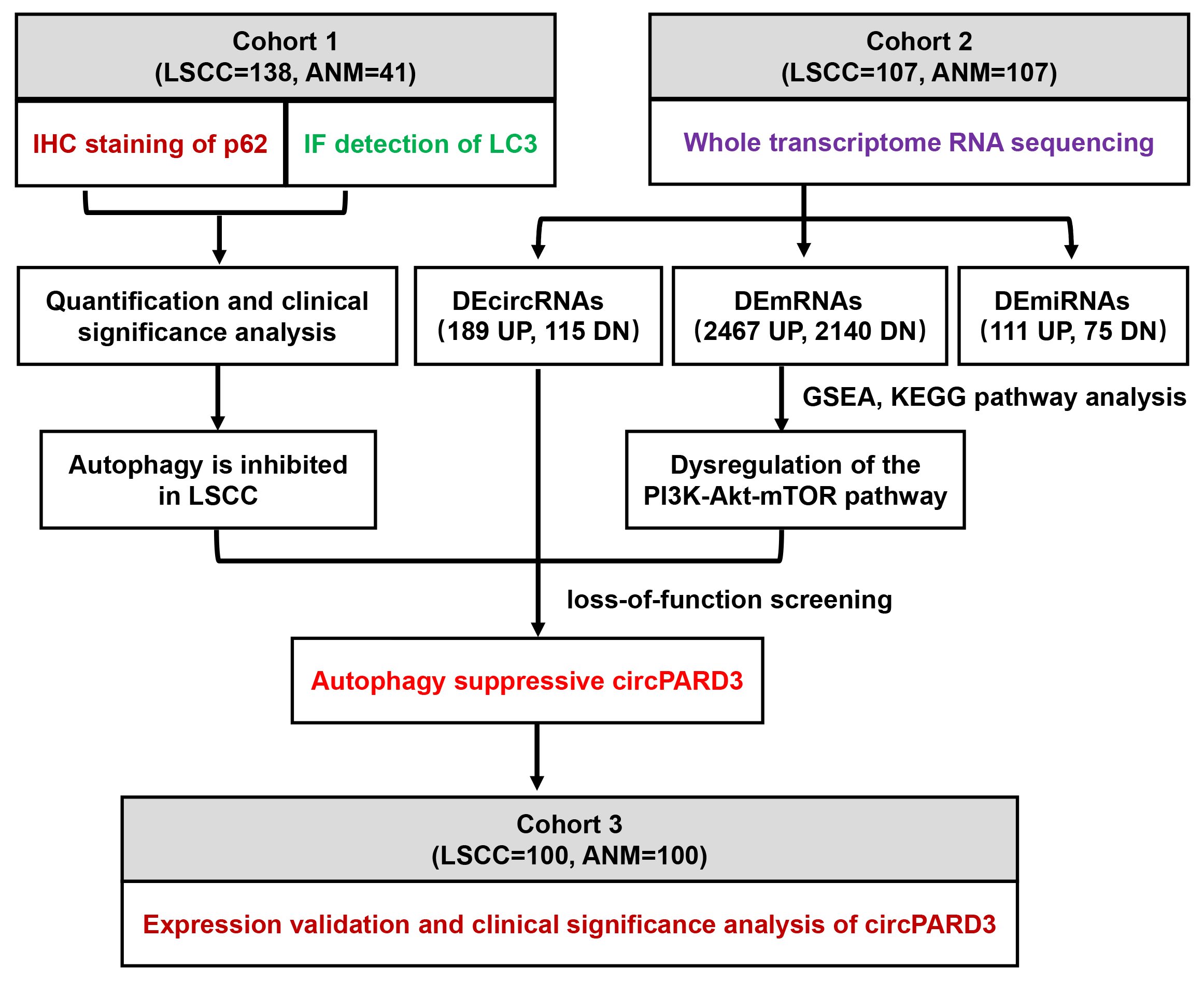

Supplement: Supplementary file 3 — Additional file 3: Figure S1. The flow chart for screening and verifying autophagy suppressive circPARD3 in LSCC. Figure S2. FD-LSC-1 and Tu 177 cells were transfected with Cy3 labeled si-NC (si-NC-Cy3), NC mimics (NC mimics-Cy3), or NC inhibitor (NC inhibitor-Cy3) for 48 h. Nuclei were stained with DAPI (blue). Transfection efficiency was evaluated by imaging with confocal microscopy. Red dot represents siRNA, miRNA mimics, or miRNA inhibitor. Scale bar, 50 μm. Figure S3. Verification of the structure features of circPARD3. a Expression of circPARD3 in FD-LSC-1 and Tu 177 cells was verified by RT-PCR. Agarose gel electrophoresis showed that divergent primers amplified circPARD3 in cDNA but not genomic DNA (gDNA). GAPDH served as a negative control. b Stability of circPARD3 and linear PARD3 mRNA was assessed by RNase R treatment and RT-PCR analysis. Figure S4. FD-LSC-1 and Tu 177 cells were infected with circPARD3 overexpression lentiviruses (circPARD3-OE) or transfected with si-circPARD3 (si-circ-1, si-circ-2) for 48 h. Expression level of linear PARD3 mRNA was determined by qPCR analysis. Error bars represent SD of three independent experiments. N.S., no significant. Figure S5. Expression levels of potential circPARD3 target miRNAs in FD-LSC-1 and Tu 177 cells with overexpression (a) or knockdown (b) of circPARD3 were determined by qPCR analysis. Error bars represent SD of three independent experiments. * P < 0.05, **P < 0.01. Figure S6. The effects of miR-145-5p on LSCC cell autophagy. a and b FD-LSC-1 and Tu 177 cells were transfected with miR-145-5p mimics (a) or inhibitor (b) for 48 h. Expression levels of p62 and LC3B were detected by western blotting. c FD-LSC-1 and Tu 177 cells were transfected with miR-145-5p mimics or inhibitor for 48 h. Autophagic flux was analyzed by confocal microscopy. Representative images (Top) and statistical data (Bottom) were shown. Scale bar, 25 μm. Error bars represent SD of three independent experiments. * P < 0.05, **P < 0.01 [file 12943_2020_1279_MOESM3_ESM.zip › Figure S1.tif]

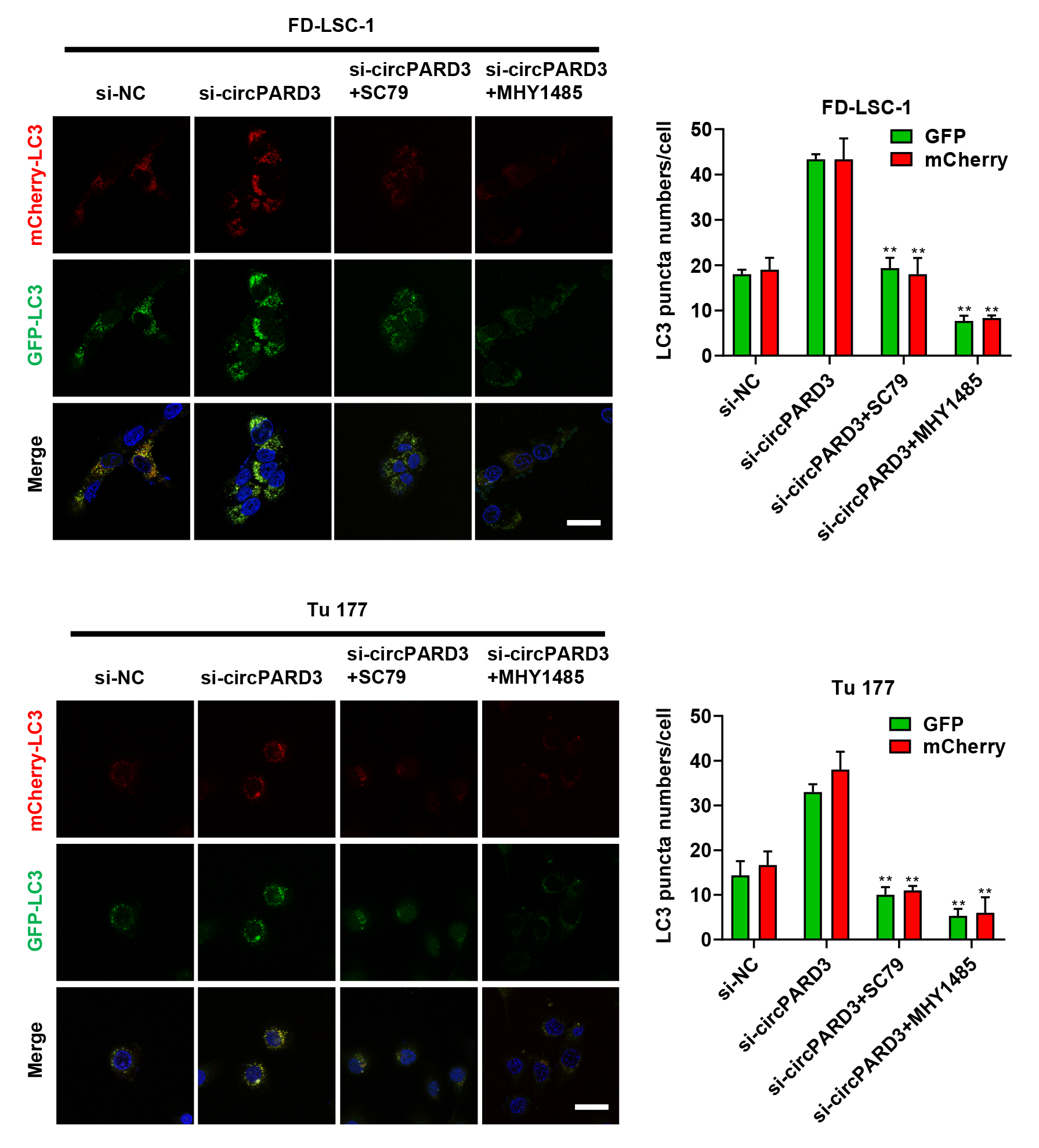

Supplement: Supplementary file 3 — Additional file 3: Figure S1. The flow chart for screening and verifying autophagy suppressive circPARD3 in LSCC. Figure S2. FD-LSC-1 and Tu 177 cells were transfected with Cy3 labeled si-NC (si-NC-Cy3), NC mimics (NC mimics-Cy3), or NC inhibitor (NC inhibitor-Cy3) for 48 h. Nuclei were stained with DAPI (blue). Transfection efficiency was evaluated by imaging with confocal microscopy. Red dot represents siRNA, miRNA mimics, or miRNA inhibitor. Scale bar, 50 μm. Figure S3. Verification of the structure features of circPARD3. a Expression of circPARD3 in FD-LSC-1 and Tu 177 cells was verified by RT-PCR. Agarose gel electrophoresis showed that divergent primers amplified circPARD3 in cDNA but not genomic DNA (gDNA). GAPDH served as a negative control. b Stability of circPARD3 and linear PARD3 mRNA was assessed by RNase R treatment and RT-PCR analysis. Figure S4. FD-LSC-1 and Tu 177 cells were infected with circPARD3 overexpression lentiviruses (circPARD3-OE) or transfected with si-circPARD3 (si-circ-1, si-circ-2) for 48 h. Expression level of linear PARD3 mRNA was determined by qPCR analysis. Error bars represent SD of three independent experiments. N.S., no significant. Figure S5. Expression levels of potential circPARD3 target miRNAs in FD-LSC-1 and Tu 177 cells with overexpression (a) or knockdown (b) of circPARD3 were determined by qPCR analysis. Error bars represent SD of three independent experiments. * P < 0.05, **P < 0.01. Figure S6. The effects of miR-145-5p on LSCC cell autophagy. a and b FD-LSC-1 and Tu 177 cells were transfected with miR-145-5p mimics (a) or inhibitor (b) for 48 h. Expression levels of p62 and LC3B were detected by western blotting. c FD-LSC-1 and Tu 177 cells were transfected with miR-145-5p mimics or inhibitor for 48 h. Autophagic flux was analyzed by confocal microscopy. Representative images (Top) and statistical data (Bottom) were shown. Scale bar, 25 μm. Error bars represent SD of three independent experiments. * P < 0.05, **P < 0.01 [file 12943_2020_1279_MOESM3_ESM.zip › Figure S10.tif]

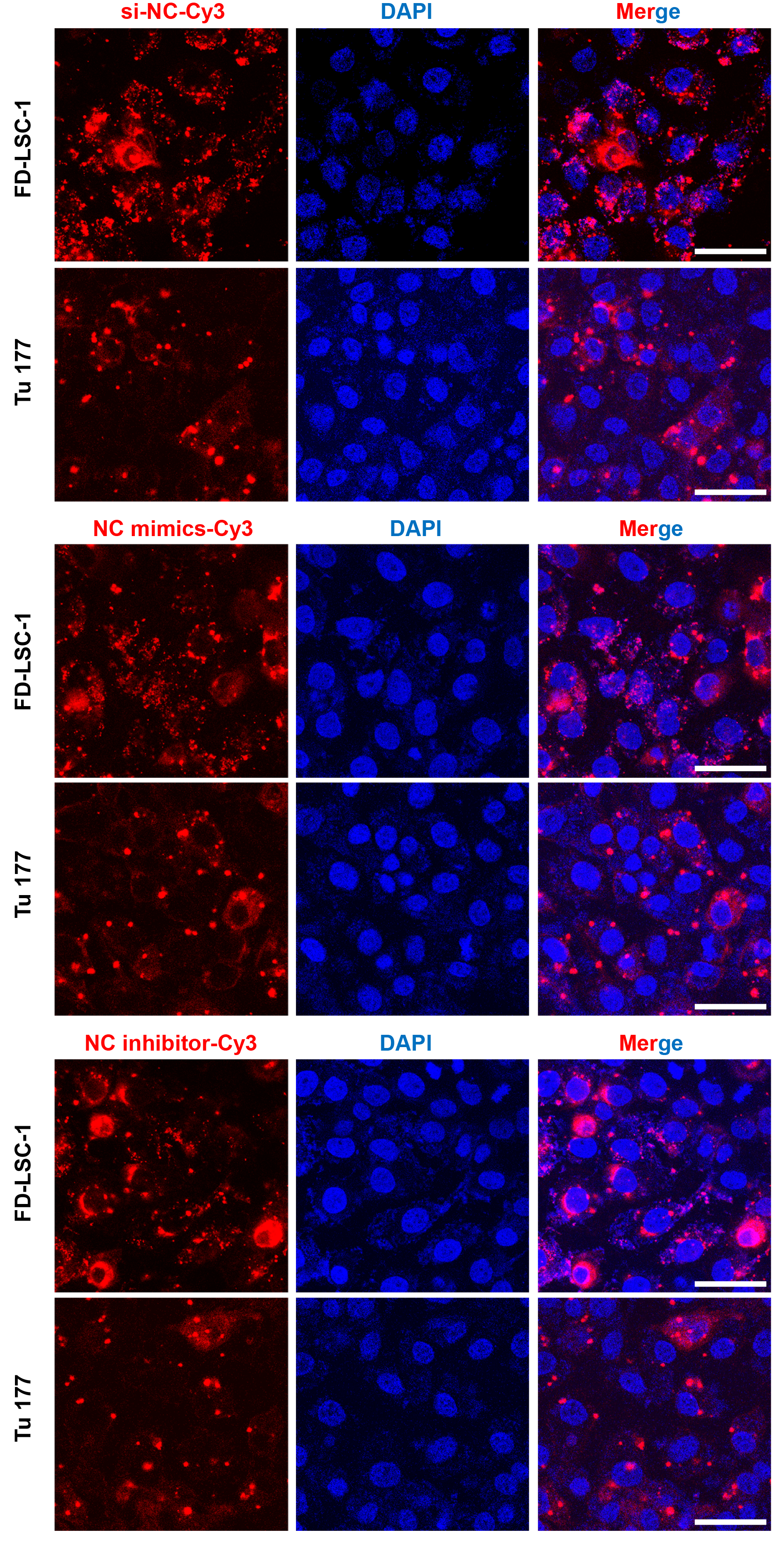

Supplement: Supplementary file 3 — Additional file 3: Figure S1. The flow chart for screening and verifying autophagy suppressive circPARD3 in LSCC. Figure S2. FD-LSC-1 and Tu 177 cells were transfected with Cy3 labeled si-NC (si-NC-Cy3), NC mimics (NC mimics-Cy3), or NC inhibitor (NC inhibitor-Cy3) for 48 h. Nuclei were stained with DAPI (blue). Transfection efficiency was evaluated by imaging with confocal microscopy. Red dot represents siRNA, miRNA mimics, or miRNA inhibitor. Scale bar, 50 μm. Figure S3. Verification of the structure features of circPARD3. a Expression of circPARD3 in FD-LSC-1 and Tu 177 cells was verified by RT-PCR. Agarose gel electrophoresis showed that divergent primers amplified circPARD3 in cDNA but not genomic DNA (gDNA). GAPDH served as a negative control. b Stability of circPARD3 and linear PARD3 mRNA was assessed by RNase R treatment and RT-PCR analysis. Figure S4. FD-LSC-1 and Tu 177 cells were infected with circPARD3 overexpression lentiviruses (circPARD3-OE) or transfected with si-circPARD3 (si-circ-1, si-circ-2) for 48 h. Expression level of linear PARD3 mRNA was determined by qPCR analysis. Error bars represent SD of three independent experiments. N.S., no significant. Figure S5. Expression levels of potential circPARD3 target miRNAs in FD-LSC-1 and Tu 177 cells with overexpression (a) or knockdown (b) of circPARD3 were determined by qPCR analysis. Error bars represent SD of three independent experiments. * P < 0.05, **P < 0.01. Figure S6. The effects of miR-145-5p on LSCC cell autophagy. a and b FD-LSC-1 and Tu 177 cells were transfected with miR-145-5p mimics (a) or inhibitor (b) for 48 h. Expression levels of p62 and LC3B were detected by western blotting. c FD-LSC-1 and Tu 177 cells were transfected with miR-145-5p mimics or inhibitor for 48 h. Autophagic flux was analyzed by confocal microscopy. Representative images (Top) and statistical data (Bottom) were shown. Scale bar, 25 μm. Error bars represent SD of three independent experiments. * P < 0.05, **P < 0.01 [file 12943_2020_1279_MOESM3_ESM.zip › Figure S2.tif]

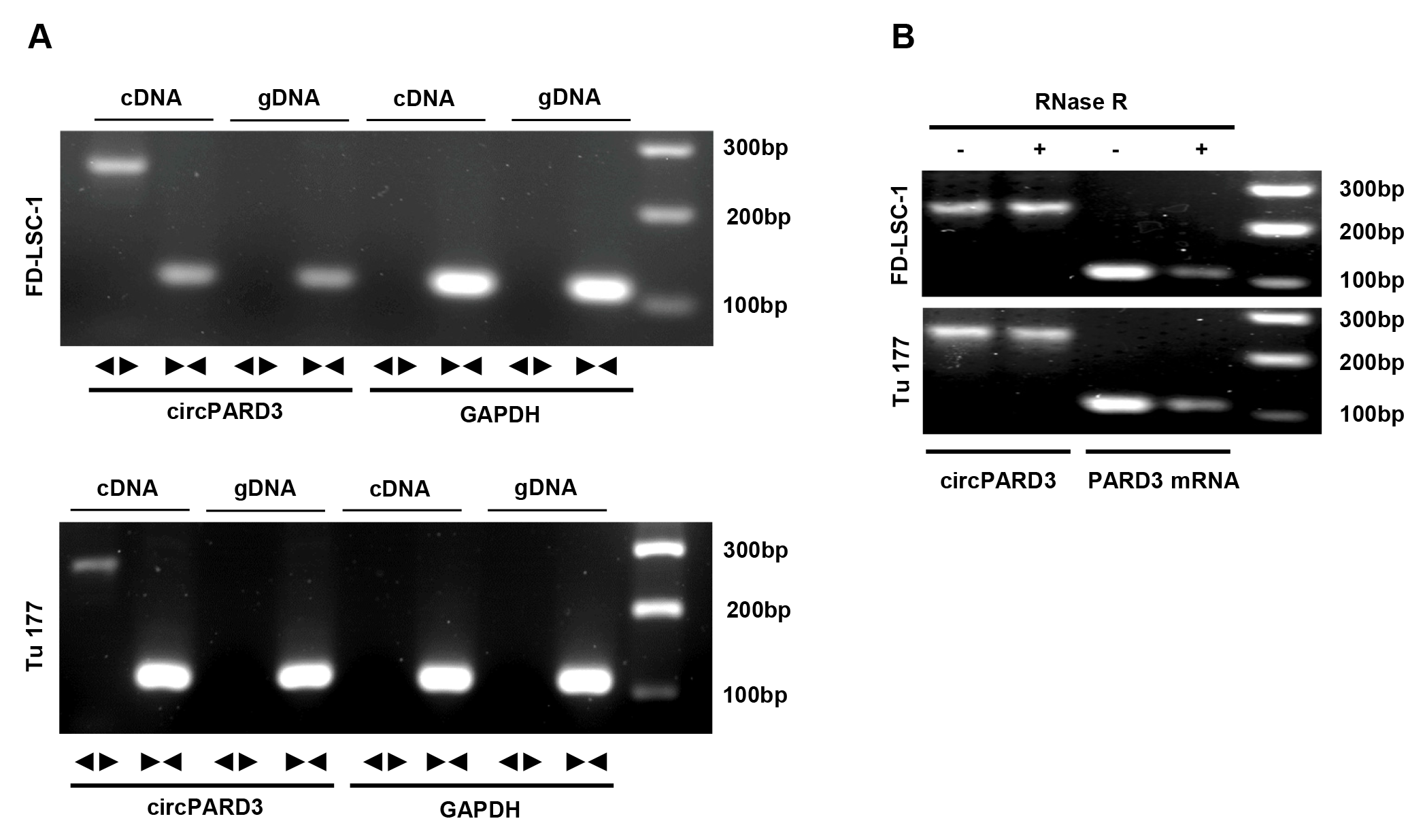

Supplement: Supplementary file 3 — Additional file 3: Figure S1. The flow chart for screening and verifying autophagy suppressive circPARD3 in LSCC. Figure S2. FD-LSC-1 and Tu 177 cells were transfected with Cy3 labeled si-NC (si-NC-Cy3), NC mimics (NC mimics-Cy3), or NC inhibitor (NC inhibitor-Cy3) for 48 h. Nuclei were stained with DAPI (blue). Transfection efficiency was evaluated by imaging with confocal microscopy. Red dot represents siRNA, miRNA mimics, or miRNA inhibitor. Scale bar, 50 μm. Figure S3. Verification of the structure features of circPARD3. a Expression of circPARD3 in FD-LSC-1 and Tu 177 cells was verified by RT-PCR. Agarose gel electrophoresis showed that divergent primers amplified circPARD3 in cDNA but not genomic DNA (gDNA). GAPDH served as a negative control. b Stability of circPARD3 and linear PARD3 mRNA was assessed by RNase R treatment and RT-PCR analysis. Figure S4. FD-LSC-1 and Tu 177 cells were infected with circPARD3 overexpression lentiviruses (circPARD3-OE) or transfected with si-circPARD3 (si-circ-1, si-circ-2) for 48 h. Expression level of linear PARD3 mRNA was determined by qPCR analysis. Error bars represent SD of three independent experiments. N.S., no significant. Figure S5. Expression levels of potential circPARD3 target miRNAs in FD-LSC-1 and Tu 177 cells with overexpression (a) or knockdown (b) of circPARD3 were determined by qPCR analysis. Error bars represent SD of three independent experiments. * P < 0.05, **P < 0.01. Figure S6. The effects of miR-145-5p on LSCC cell autophagy. a and b FD-LSC-1 and Tu 177 cells were transfected with miR-145-5p mimics (a) or inhibitor (b) for 48 h. Expression levels of p62 and LC3B were detected by western blotting. c FD-LSC-1 and Tu 177 cells were transfected with miR-145-5p mimics or inhibitor for 48 h. Autophagic flux was analyzed by confocal microscopy. Representative images (Top) and statistical data (Bottom) were shown. Scale bar, 25 μm. Error bars represent SD of three independent experiments. * P < 0.05, **P < 0.01 [file 12943_2020_1279_MOESM3_ESM.zip › Figure S3.tif]

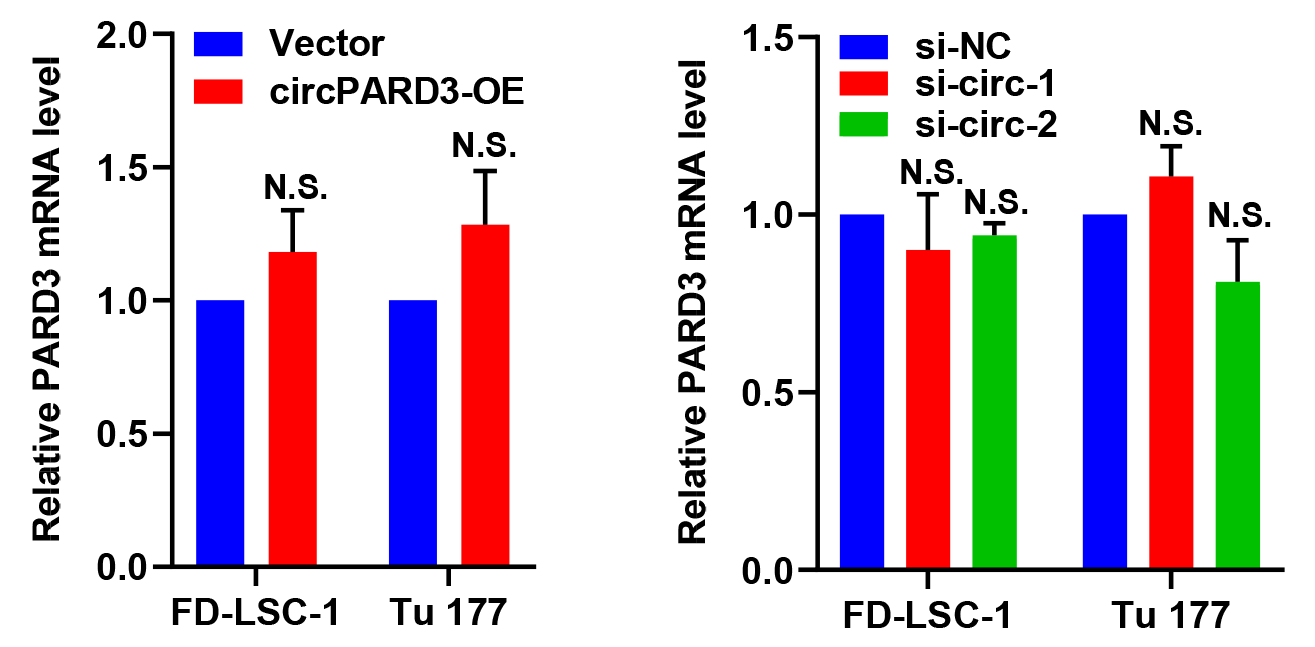

Supplement: Supplementary file 3 — Additional file 3: Figure S1. The flow chart for screening and verifying autophagy suppressive circPARD3 in LSCC. Figure S2. FD-LSC-1 and Tu 177 cells were transfected with Cy3 labeled si-NC (si-NC-Cy3), NC mimics (NC mimics-Cy3), or NC inhibitor (NC inhibitor-Cy3) for 48 h. Nuclei were stained with DAPI (blue). Transfection efficiency was evaluated by imaging with confocal microscopy. Red dot represents siRNA, miRNA mimics, or miRNA inhibitor. Scale bar, 50 μm. Figure S3. Verification of the structure features of circPARD3. a Expression of circPARD3 in FD-LSC-1 and Tu 177 cells was verified by RT-PCR. Agarose gel electrophoresis showed that divergent primers amplified circPARD3 in cDNA but not genomic DNA (gDNA). GAPDH served as a negative control. b Stability of circPARD3 and linear PARD3 mRNA was assessed by RNase R treatment and RT-PCR analysis. Figure S4. FD-LSC-1 and Tu 177 cells were infected with circPARD3 overexpression lentiviruses (circPARD3-OE) or transfected with si-circPARD3 (si-circ-1, si-circ-2) for 48 h. Expression level of linear PARD3 mRNA was determined by qPCR analysis. Error bars represent SD of three independent experiments. N.S., no significant. Figure S5. Expression levels of potential circPARD3 target miRNAs in FD-LSC-1 and Tu 177 cells with overexpression (a) or knockdown (b) of circPARD3 were determined by qPCR analysis. Error bars represent SD of three independent experiments. * P < 0.05, **P < 0.01. Figure S6. The effects of miR-145-5p on LSCC cell autophagy. a and b FD-LSC-1 and Tu 177 cells were transfected with miR-145-5p mimics (a) or inhibitor (b) for 48 h. Expression levels of p62 and LC3B were detected by western blotting. c FD-LSC-1 and Tu 177 cells were transfected with miR-145-5p mimics or inhibitor for 48 h. Autophagic flux was analyzed by confocal microscopy. Representative images (Top) and statistical data (Bottom) were shown. Scale bar, 25 μm. Error bars represent SD of three independent experiments. * P < 0.05, **P < 0.01 [file 12943_2020_1279_MOESM3_ESM.zip › Figure S4.tif]

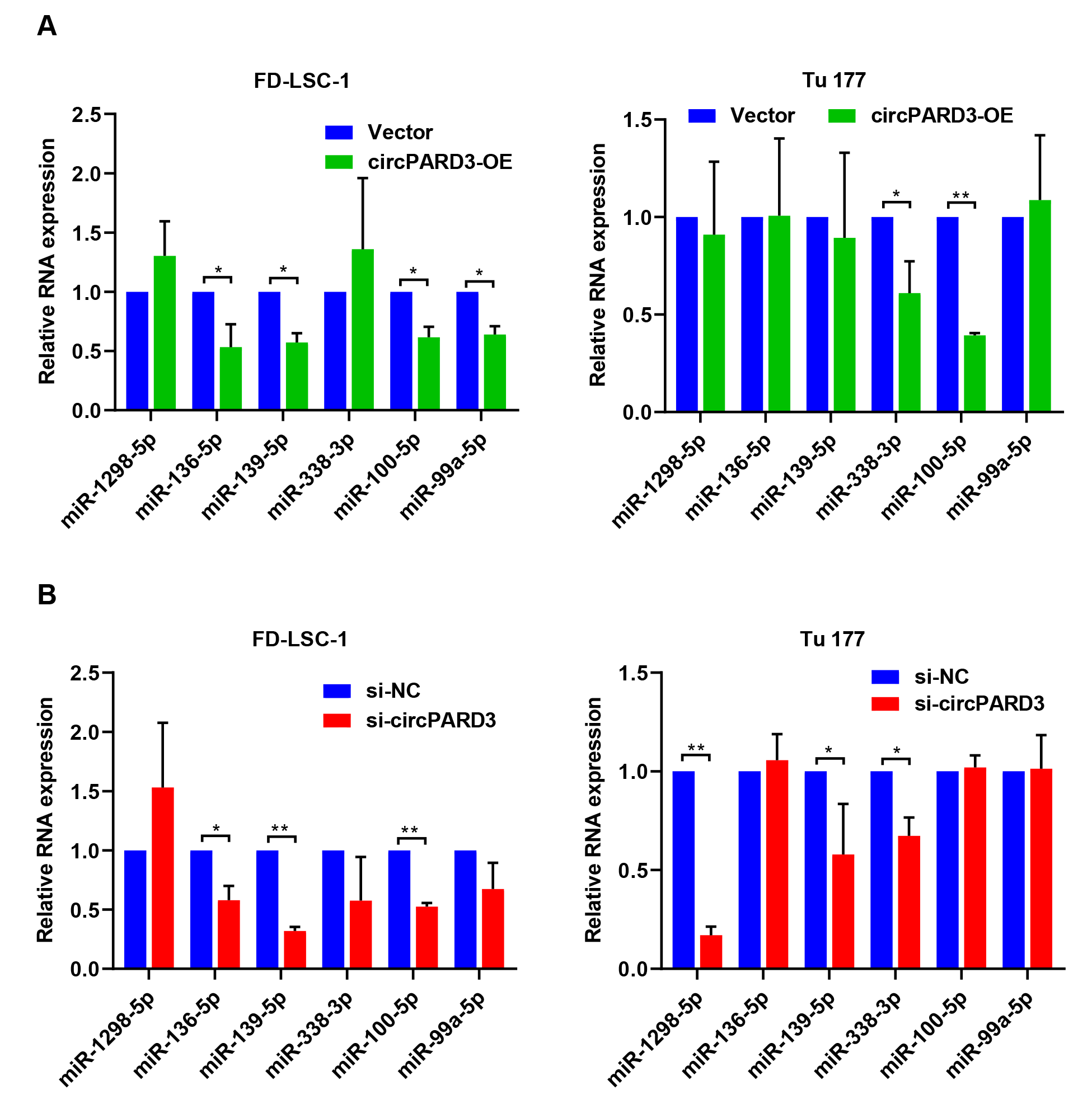

Supplement: Supplementary file 3 — Additional file 3: Figure S1. The flow chart for screening and verifying autophagy suppressive circPARD3 in LSCC. Figure S2. FD-LSC-1 and Tu 177 cells were transfected with Cy3 labeled si-NC (si-NC-Cy3), NC mimics (NC mimics-Cy3), or NC inhibitor (NC inhibitor-Cy3) for 48 h. Nuclei were stained with DAPI (blue). Transfection efficiency was evaluated by imaging with confocal microscopy. Red dot represents siRNA, miRNA mimics, or miRNA inhibitor. Scale bar, 50 μm. Figure S3. Verification of the structure features of circPARD3. a Expression of circPARD3 in FD-LSC-1 and Tu 177 cells was verified by RT-PCR. Agarose gel electrophoresis showed that divergent primers amplified circPARD3 in cDNA but not genomic DNA (gDNA). GAPDH served as a negative control. b Stability of circPARD3 and linear PARD3 mRNA was assessed by RNase R treatment and RT-PCR analysis. Figure S4. FD-LSC-1 and Tu 177 cells were infected with circPARD3 overexpression lentiviruses (circPARD3-OE) or transfected with si-circPARD3 (si-circ-1, si-circ-2) for 48 h. Expression level of linear PARD3 mRNA was determined by qPCR analysis. Error bars represent SD of three independent experiments. N.S., no significant. Figure S5. Expression levels of potential circPARD3 target miRNAs in FD-LSC-1 and Tu 177 cells with overexpression (a) or knockdown (b) of circPARD3 were determined by qPCR analysis. Error bars represent SD of three independent experiments. * P < 0.05, **P < 0.01. Figure S6. The effects of miR-145-5p on LSCC cell autophagy. a and b FD-LSC-1 and Tu 177 cells were transfected with miR-145-5p mimics (a) or inhibitor (b) for 48 h. Expression levels of p62 and LC3B were detected by western blotting. c FD-LSC-1 and Tu 177 cells were transfected with miR-145-5p mimics or inhibitor for 48 h. Autophagic flux was analyzed by confocal microscopy. Representative images (Top) and statistical data (Bottom) were shown. Scale bar, 25 μm. Error bars represent SD of three independent experiments. * P < 0.05, **P < 0.01 [file 12943_2020_1279_MOESM3_ESM.zip › Figure S5.tif]

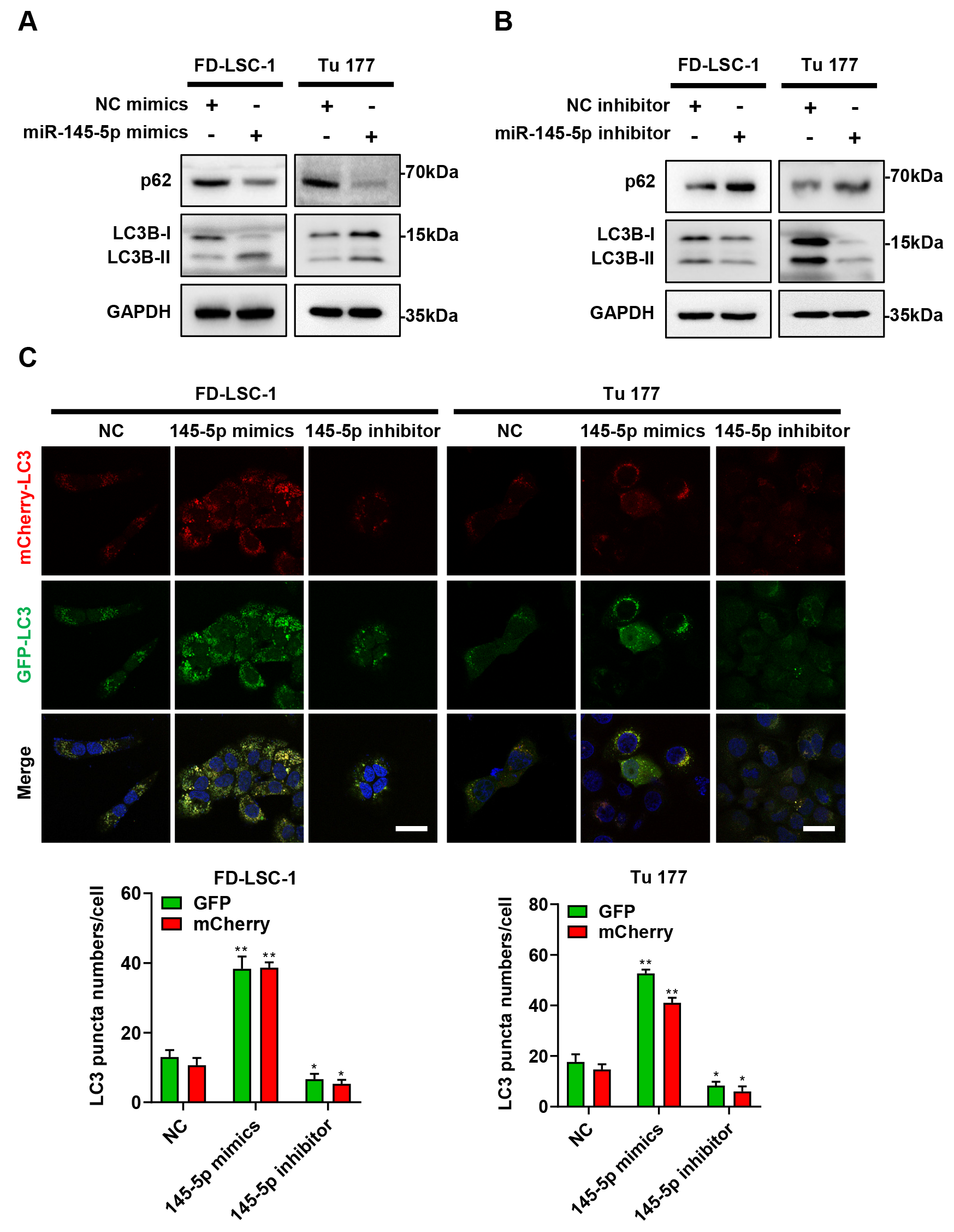

Supplement: Supplementary file 3 — Additional file 3: Figure S1. The flow chart for screening and verifying autophagy suppressive circPARD3 in LSCC. Figure S2. FD-LSC-1 and Tu 177 cells were transfected with Cy3 labeled si-NC (si-NC-Cy3), NC mimics (NC mimics-Cy3), or NC inhibitor (NC inhibitor-Cy3) for 48 h. Nuclei were stained with DAPI (blue). Transfection efficiency was evaluated by imaging with confocal microscopy. Red dot represents siRNA, miRNA mimics, or miRNA inhibitor. Scale bar, 50 μm. Figure S3. Verification of the structure features of circPARD3. a Expression of circPARD3 in FD-LSC-1 and Tu 177 cells was verified by RT-PCR. Agarose gel electrophoresis showed that divergent primers amplified circPARD3 in cDNA but not genomic DNA (gDNA). GAPDH served as a negative control. b Stability of circPARD3 and linear PARD3 mRNA was assessed by RNase R treatment and RT-PCR analysis. Figure S4. FD-LSC-1 and Tu 177 cells were infected with circPARD3 overexpression lentiviruses (circPARD3-OE) or transfected with si-circPARD3 (si-circ-1, si-circ-2) for 48 h. Expression level of linear PARD3 mRNA was determined by qPCR analysis. Error bars represent SD of three independent experiments. N.S., no significant. Figure S5. Expression levels of potential circPARD3 target miRNAs in FD-LSC-1 and Tu 177 cells with overexpression (a) or knockdown (b) of circPARD3 were determined by qPCR analysis. Error bars represent SD of three independent experiments. * P < 0.05, **P < 0.01. Figure S6. The effects of miR-145-5p on LSCC cell autophagy. a and b FD-LSC-1 and Tu 177 cells were transfected with miR-145-5p mimics (a) or inhibitor (b) for 48 h. Expression levels of p62 and LC3B were detected by western blotting. c FD-LSC-1 and Tu 177 cells were transfected with miR-145-5p mimics or inhibitor for 48 h. Autophagic flux was analyzed by confocal microscopy. Representative images (Top) and statistical data (Bottom) were shown. Scale bar, 25 μm. Error bars represent SD of three independent experiments. * P < 0.05, **P < 0.01 [file 12943_2020_1279_MOESM3_ESM.zip › Figure S6.tif]

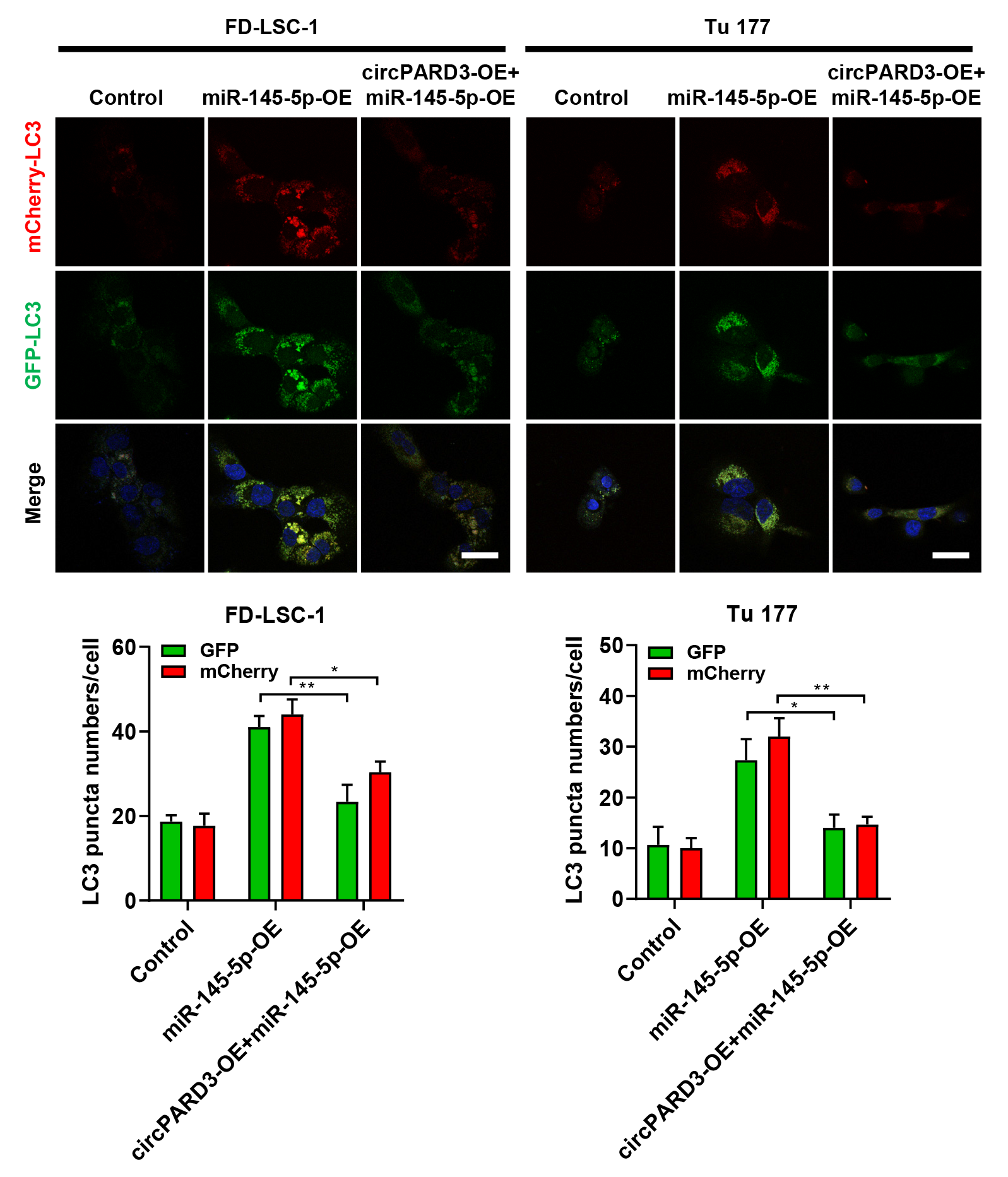

Supplement: Supplementary file 3 — Additional file 3: Figure S1. The flow chart for screening and verifying autophagy suppressive circPARD3 in LSCC. Figure S2. FD-LSC-1 and Tu 177 cells were transfected with Cy3 labeled si-NC (si-NC-Cy3), NC mimics (NC mimics-Cy3), or NC inhibitor (NC inhibitor-Cy3) for 48 h. Nuclei were stained with DAPI (blue). Transfection efficiency was evaluated by imaging with confocal microscopy. Red dot represents siRNA, miRNA mimics, or miRNA inhibitor. Scale bar, 50 μm. Figure S3. Verification of the structure features of circPARD3. a Expression of circPARD3 in FD-LSC-1 and Tu 177 cells was verified by RT-PCR. Agarose gel electrophoresis showed that divergent primers amplified circPARD3 in cDNA but not genomic DNA (gDNA). GAPDH served as a negative control. b Stability of circPARD3 and linear PARD3 mRNA was assessed by RNase R treatment and RT-PCR analysis. Figure S4. FD-LSC-1 and Tu 177 cells were infected with circPARD3 overexpression lentiviruses (circPARD3-OE) or transfected with si-circPARD3 (si-circ-1, si-circ-2) for 48 h. Expression level of linear PARD3 mRNA was determined by qPCR analysis. Error bars represent SD of three independent experiments. N.S., no significant. Figure S5. Expression levels of potential circPARD3 target miRNAs in FD-LSC-1 and Tu 177 cells with overexpression (a) or knockdown (b) of circPARD3 were determined by qPCR analysis. Error bars represent SD of three independent experiments. * P < 0.05, **P < 0.01. Figure S6. The effects of miR-145-5p on LSCC cell autophagy. a and b FD-LSC-1 and Tu 177 cells were transfected with miR-145-5p mimics (a) or inhibitor (b) for 48 h. Expression levels of p62 and LC3B were detected by western blotting. c FD-LSC-1 and Tu 177 cells were transfected with miR-145-5p mimics or inhibitor for 48 h. Autophagic flux was analyzed by confocal microscopy. Representative images (Top) and statistical data (Bottom) were shown. Scale bar, 25 μm. Error bars represent SD of three independent experiments. * P < 0.05, **P < 0.01 [file 12943_2020_1279_MOESM3_ESM.zip › Figure S7.tif]

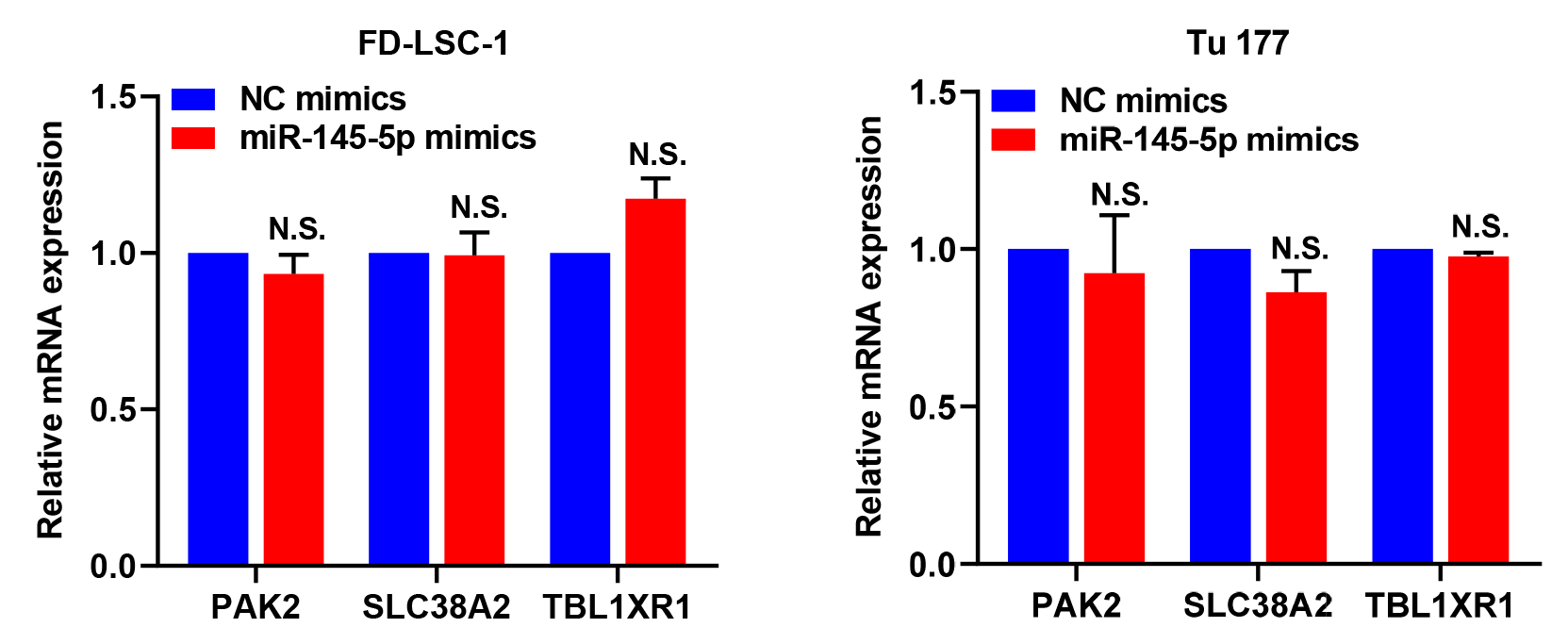

Supplement: Supplementary file 3 — Additional file 3: Figure S1. The flow chart for screening and verifying autophagy suppressive circPARD3 in LSCC. Figure S2. FD-LSC-1 and Tu 177 cells were transfected with Cy3 labeled si-NC (si-NC-Cy3), NC mimics (NC mimics-Cy3), or NC inhibitor (NC inhibitor-Cy3) for 48 h. Nuclei were stained with DAPI (blue). Transfection efficiency was evaluated by imaging with confocal microscopy. Red dot represents siRNA, miRNA mimics, or miRNA inhibitor. Scale bar, 50 μm. Figure S3. Verification of the structure features of circPARD3. a Expression of circPARD3 in FD-LSC-1 and Tu 177 cells was verified by RT-PCR. Agarose gel electrophoresis showed that divergent primers amplified circPARD3 in cDNA but not genomic DNA (gDNA). GAPDH served as a negative control. b Stability of circPARD3 and linear PARD3 mRNA was assessed by RNase R treatment and RT-PCR analysis. Figure S4. FD-LSC-1 and Tu 177 cells were infected with circPARD3 overexpression lentiviruses (circPARD3-OE) or transfected with si-circPARD3 (si-circ-1, si-circ-2) for 48 h. Expression level of linear PARD3 mRNA was determined by qPCR analysis. Error bars represent SD of three independent experiments. N.S., no significant. Figure S5. Expression levels of potential circPARD3 target miRNAs in FD-LSC-1 and Tu 177 cells with overexpression (a) or knockdown (b) of circPARD3 were determined by qPCR analysis. Error bars represent SD of three independent experiments. * P < 0.05, **P < 0.01. Figure S6. The effects of miR-145-5p on LSCC cell autophagy. a and b FD-LSC-1 and Tu 177 cells were transfected with miR-145-5p mimics (a) or inhibitor (b) for 48 h. Expression levels of p62 and LC3B were detected by western blotting. c FD-LSC-1 and Tu 177 cells were transfected with miR-145-5p mimics or inhibitor for 48 h. Autophagic flux was analyzed by confocal microscopy. Representative images (Top) and statistical data (Bottom) were shown. Scale bar, 25 μm. Error bars represent SD of three independent experiments. * P < 0.05, **P < 0.01 [file 12943_2020_1279_MOESM3_ESM.zip › Figure S8.tif]

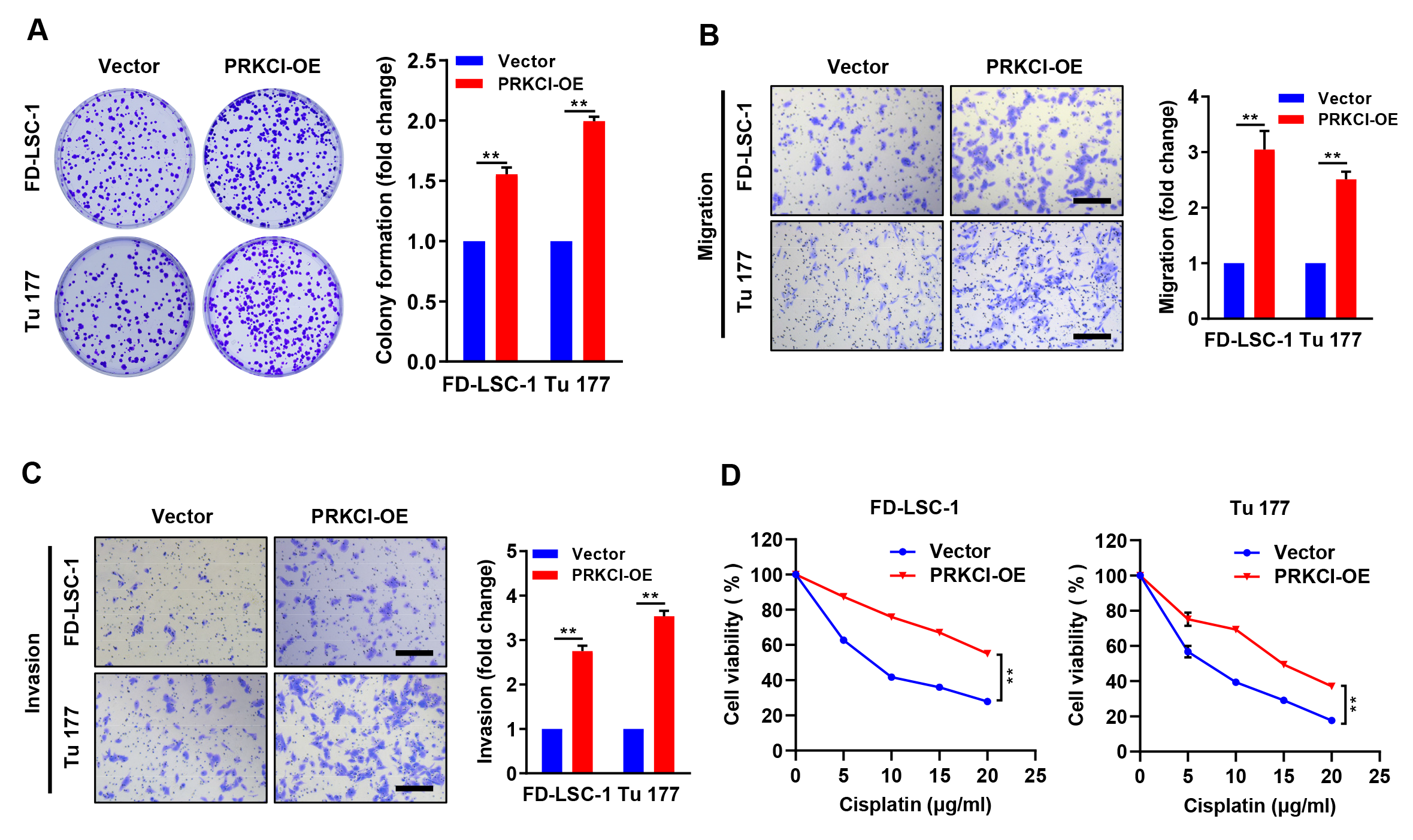

Supplement: Supplementary file 3 — Additional file 3: Figure S1. The flow chart for screening and verifying autophagy suppressive circPARD3 in LSCC. Figure S2. FD-LSC-1 and Tu 177 cells were transfected with Cy3 labeled si-NC (si-NC-Cy3), NC mimics (NC mimics-Cy3), or NC inhibitor (NC inhibitor-Cy3) for 48 h. Nuclei were stained with DAPI (blue). Transfection efficiency was evaluated by imaging with confocal microscopy. Red dot represents siRNA, miRNA mimics, or miRNA inhibitor. Scale bar, 50 μm. Figure S3. Verification of the structure features of circPARD3. a Expression of circPARD3 in FD-LSC-1 and Tu 177 cells was verified by RT-PCR. Agarose gel electrophoresis showed that divergent primers amplified circPARD3 in cDNA but not genomic DNA (gDNA). GAPDH served as a negative control. b Stability of circPARD3 and linear PARD3 mRNA was assessed by RNase R treatment and RT-PCR analysis. Figure S4. FD-LSC-1 and Tu 177 cells were infected with circPARD3 overexpression lentiviruses (circPARD3-OE) or transfected with si-circPARD3 (si-circ-1, si-circ-2) for 48 h. Expression level of linear PARD3 mRNA was determined by qPCR analysis. Error bars represent SD of three independent experiments. N.S., no significant. Figure S5. Expression levels of potential circPARD3 target miRNAs in FD-LSC-1 and Tu 177 cells with overexpression (a) or knockdown (b) of circPARD3 were determined by qPCR analysis. Error bars represent SD of three independent experiments. * P < 0.05, **P < 0.01. Figure S6. The effects of miR-145-5p on LSCC cell autophagy. a and b FD-LSC-1 and Tu 177 cells were transfected with miR-145-5p mimics (a) or inhibitor (b) for 48 h. Expression levels of p62 and LC3B were detected by western blotting. c FD-LSC-1 and Tu 177 cells were transfected with miR-145-5p mimics or inhibitor for 48 h. Autophagic flux was analyzed by confocal microscopy. Representative images (Top) and statistical data (Bottom) were shown. Scale bar, 25 μm. Error bars represent SD of three independent experiments. * P < 0.05, **P < 0.01 [file 12943_2020_1279_MOESM3_ESM.zip › Figure S9.tif]
